# Trichlorobenzene-substituted azaaryl compounds as novel FGFR inhibitors exhibiting potent antitumor activity in bladder cancer cells *in vitro* and *in vivo*

**DOI:** 10.18632/oncotarget.8380

**Published:** 2016-03-25

**Authors:** Chun-Han Chen, Yi-Min Liu, Shiow-Lin Pan, Yun-Ru Liu, Jing-Ping Liou, Yun Yen

**Affiliations:** ^1^ Department of Pharmacology, School of Medicine, College of Medicine, Taipei Medical University, Taipei, Taiwan; ^2^ College of Pharmacy, Taipei Medical University, Taipei, Taiwan; ^3^ The Ph.D. Program for Cancer Biology and Drug Discovery, College of Medical Science and Technology, Taipei Medical University, Taipei, Taiwan; ^4^ Joint Biobank, Office of Human Research, Taipei Medical University, Taipei, Taiwan

**Keywords:** FGFR, bladder cancer, autophagy, cell cycle, FGFR3-TACC3

## Abstract

In the present study, we examined the antitumor activity of a series of trichlorobenzene-substituted azaaryl compounds and identified MPT0L145 as a novel FGFR inhibitor with better selectivity for FGFR1, 2 and 3. It was preferentially effective in FGFR-activated cancer cells, including bladder cancer cell lines expressing FGFR3-TACC3 fusion proteins (RT-112, RT-4). MPT0L145 decreased the phosphorylation of FGFR1, FGFR3 and their downstream proteins (FRS2, ERK and Akt). Mechanistically, cDNA microarray analysis revealed that MPT0L145 decreased genes associated cell cycle progression, and increased genes associated with autophagy pathway. Accordingly, the data revealed that MPT0L145 induced G_0_/G_1_ cell cycle arrest and decreased protein levels of cyclin E. Moreover, we provided the evidence that autophagy contributes to FGFR inhibitor-related cell death. Finally, MPT0L145 exhibited comparable antitumor activity to cisplatin with better safety in a RT-112 xenograft model. Taken together, these findings support the utility of MPT0L145 as a novel FGFR inhibitor, providing a strong rationale for further evaluation of this compound as a therapeutic agent for bladder cancers.

## INTRODUCTION

Fibroblast growth factors (FGF) and its receptors (FGFR) play a crucial role in regulating key physiologic processes, such as proliferation, migration, differentiation, survival, embryogenesis, angiogenesis and wound healing in adults. The fibroblast growth factor receptor (FGFR) family of receptor tyrosine kinases includes four highly conserved isoforms (FGFR1-4), which are differentially activated by 18 different FGF ligands. FGFR5 (FGFRL1) reportedly binds FGFs, but lacks the tyrosine kinase domain, and is proposed to serve as a negatively regulator [1]. Moreover, deregulation of FGF signaling is associated with the pathogenesis of developmental disorders and cancers originating from different tissue types [2, 3]. FGFR1 signaling has been implicated in tumor progression, based on its amplification in 20% squamous non–small cell lung cancers (NSCLC) and in 10% breast cancers [4, 5]. FGFR2-activating mutations have been identified in 12% endometrial carcinomas and ~10% gastric cancers shown to harbor FGFR2 amplification and mutations [6, 7]. FGFR3 is aberrantly expressed in 15-20% of multiple myelomas with the t(4;14) translocation [8]. A number of rhabdomyosarcoma (7–8%) cases harboring FGFR4-activating mutations have been identified [9]. Recently, FGF19/FGFR4 was reported to cause hepatocellular carcinoma (HCC) in mice, providing further insights into the pathogenesis of the disease in humans [10, 11]. Clinical reagents that specifically target the FGF/FGFR signaling axis are thus currently under development as potential therapeutic options [12].

Bladder cancer is a common malignancy with an estimated 429,800 new cases of bladder cancer and 165,100 deaths occurred worldwide [13]. In the United States alone, 74,000 new cases of bladder cancer and 16,000 deaths are estimated in 2015 [14]. Statistically, ~70% of cases are non-muscle invasive bladder cancers (NMIBC) with high propensity of recurrence (50-70%) and five-year survival rate of about 90 %. The other 30% of cases are muscle invasive bladder cancers (MIBC), which commonly progress to metastasis with a 5-year survival of ~50% [12]. Studies over the past decade have clearly demonstrated that FGF/FGFR signaling is aberrantly altered in bladder cancers [15]. FGFR3 has been identified as a particularly rational target for bladder cancer therapy [16]. Active mutations in FGFR3 have been described in 50-60% of NMIBCs and 10-15% of MIBCs [17]. The recent identification of FGFR fusions in multiple cancer types has further provided a new class of FGFR-associated drug targets [18, 19]. Overall ~5% of tumors display chromosomal rearrangements that generate constitutively activated fusion proteins, including FGFR3-transforming acid coiled coil 3 (TACC3) resulting from 4p16.3 re-arrangement and t(4;7) that generates the FGFR3-BAI1-associated protein 2-like 1 (BAIAP2L1) fusion. In view of these aberrant alternations, FGFR3 is recognized as a unique potential therapeutic target for bladder cancer patients.

Aberrant activation of FGF/FGFR signaling makes this pathway an attractive target for the discovery of novel anticancer drugs. The most clinically advanced FGFR inhibitors to date are non-selective TKIs, such as nintedanib, lenvatinib, ponatinib, and dovitinib. These agents usually have multiple targets, including VEGFRs, PDGFRs, KIT, and FLT3. However, the lack of kinase selectivity can trigger various side- and toxic effects in clinical and preclinical studies [20]. NVP-BGJ-398 is a novel pan-selective inhibitor of FGFR currently under phase II clinical trial. The docking model suggests that selectivity for FGFR kinases is determined by the optimal fit of the 2,6- dichloro-3,5-dimethoxy-phenyl moiety to the particular shape of the ATP site back pocket [21]. In the current study, we focused on the anticancer activity of chlorobenzene-substituted azaaryl compounds with the 2,4,6-trichloro-3,5-dimethoxyphenyl moiety. MPT0L145 was identified as a novel selective pan-FGFR inhibitor with enhanced potency for FGFR-activated cancer cells and lower toxicity to normal cells. MPT0L145 exhibited inhibitory activity on the auto-phosphorylation of FGFR1 and FGFR3, as well as phosphorylation of their downstream proteins, FRS2, ERK and AKT. The pharmacological mechanisms of MPT0L145 were also elucidated in current study. Moreover, MPT0L145 displayed comparable antitumor activity to cisplatin, alone with better safety in a RT-112 xenograft model *in vivo*. These findings collectively support the further development of MPT0L145 as a potentially safe and effective therapeutic agent for FGFR-dysregulated cancers.

## RESULTS

### Synthesis and screening of trichlorobenzene-substituted azaaryl compounds

**Figure 1:**
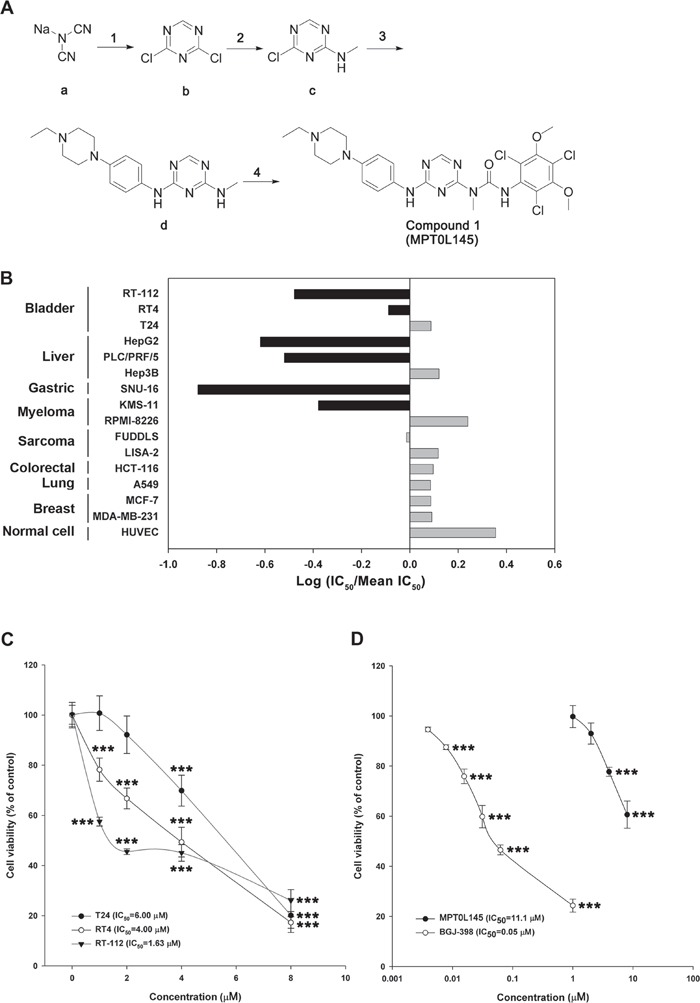
MPT0L145 inhibits FGFR signaling and exerts anti-growth effects on FGFR-activated cancer cell lines **A.** Synthesis of compound 1 (MPT0L145). Reagents and condition. (1) conc. HCl (aq.), H_2_O, -78°C then POCl_3_, DMF, DCM, 0°C to rt; (2) 2 M Methylamine in THF, IPA, rt; (3) 4-(4-Ethyl-piperazin-1-yl)-phenylamine, AcOH/H_2_O, reflux; (4) 2,4,6-Trichloro-3,5-dimethoxy-phenylamine, triphosgene, p-dioxane, toluene, reflux then toluene, reflux. Abbreviations: DMF, N, N-dimethylformamide; DCM, dichloromethane; THF, tetrahydrofuran; IPA, isopropyl alcohol; AcOH, acetic acid. The details are described in Supplementary Methods. **B.** The anti-growth activity of MPT0L145 was examined in a panel of 15 cancer cell lines and normal HUVEC cells. Data are expressed as Log (IC_50_/Mean IC_50_). Black bar depicts cell lines in which FGFR signaling is reportedly activated. **C.** Effects of MPT0L145 on the viability of bladder cancer cells. RT-112, RT4 and T24 cells were treated with the indicated concentrations of MPT0L145 for 72 h. Cell viability was assessed the MTT assay. Data are expressed as means ± S.D. (***P < 0.001 compare to control group) **D.** MPT0L145 has less toxicity to normal cells. HUVECs were treated with indicated concentrations of MPT0L145 for 72 hours and viability was examined by MTT assay. Data are expressed as means ± S.D. (***P < 0.001 compare to control group)

The synthetic routes for generating compounds are depicted in Supplementary Figure S1, and the details provided in Supplementary Methods. To examine the potential anticancer effects of trichlorobenzene-substituted azaaryl compounds, we performed drug screening with the MTT assay in several cancer cell lines. In the RT-112 cell line, compounds suppressing more than 50% viability at a concentration of 10 μM were selected and further tested on other cancer cells at a concentration of 5 μM. MPT0L145 was the most potent among the drugs examined in inhibiting cell growth of RT-112 and PLC/PRF/5 cells, and to a lesser extent, SNU-16 cells (Supplementary Table S1), and was therefore selected for further assessment of anticancer activity and pharmacological mechanisms. The synthetic pathway of MPT0L145 (compound 1) is depicted in Figure 1A. Compound (b) was cyclized from the commercial available sodium dicyanamide (a) under acid condition, with 13% yield. Aminomethylation of compound (c) was achieved by reaction of methylamine with compound (b), leading to 72% yield. Direct nucleophilic substitution by aniline with the substituted piperazine group generated compound (d) with 33% yield. Compound 1 (MPT0L145) with urea linkage was generated (16% yield) by treatment with 2,4,6-trichloro-3,5-dimethoxy-phenylamine and triphosgene to generate the intermediate, followed by compound (d) attached.

### MPT0L145 is a selective pan-FGFR inhibitor that exhibits selectivity in cancer cells displaying FGFR activation

To examine the selectivity of MPT0L145, *in vitro* assays were conducted against a panel of protein kinases. MPT0L145 displayed potent inhibitory activity on FGFR1 to FGFR3, and to a lesser extent FGFR4 (Supplementary Table S2), as well as selectivity over other kinases, including EGFR, Erbb2, IGF1R, KIT, FLT3 and VEGFR2. We also examined cytotoxic effects of MPT0L145 in a panel of 15 cell lines comprising multiple tumor types (bladder, liver, gastric, myeloma, sarcoma, colorectal, lung, breast) as well as normal cells (HUVEC). Figure 1B represents the fold change of IC_50_ in each cell line from the mean IC_50_ of all cell lines. The data suggested that MPT0L145 exhibits higher potency in the cells reportedly expressing dysregulated-FGFRs. The mean IC_50_ values in FGFR-dependent versus FGFR-independent cells were 1.83 μM and 6.74 μM, respectively (Supplementary Table S3). These data collectively suggest that MPT0L145 is a novel pan-selective FGFR inhibitor with higher potency in cancer cells displaying FGFR activation.

### Anti-growth activity of MPT0L145 in bladder cancer cells

**Figure 2:**
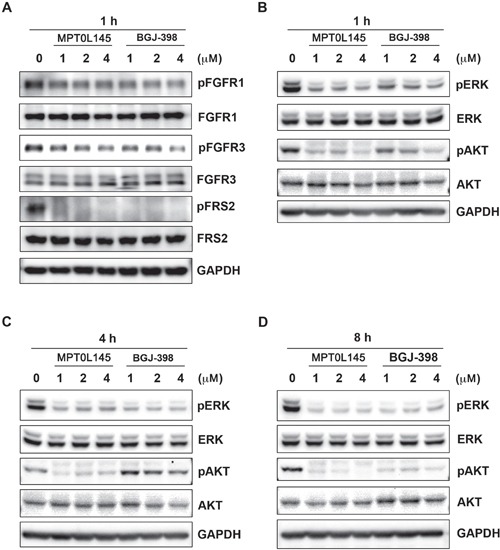
Inhibition of FGFR signaling by MPT0L145 in RT-112 cells **A.** RT-112 cells were treated with the indicated concentrations of MPT0L145 and BGJ-398 for 1 h and the levels of phosphorylated FGFR1, FGFR3 and FRS2 were detected via western blot. **(B–D)** Effects of MPT0L145 on FGFR downstream signaling. Cells were treated with the indicated concentrations of MPT0L145 or BGJ-398 for 1 h B. 4 h C. and 8 h D. Protein lysates were subjected to western blot analysis with the indicated antibodies.

Activating mutations, gene fusion and overexpression of FGFR3 in bladder cancer have been documented [15], indicating that bladder cancer is a promising indication for the discovery of novel FGFR inhibitors. We examined the anti-growth effects of MPT0L145 on bladder cancer cells with different genetic background of FGFR3. Cells with the FGFR3-TACC3 fusion (RT-112, RT4) were more sensitive to MPT0L145 than those with normal FGFR3 status (T24) (Figure 1C). Notably, MPT0L145 induced significantly lower toxicity in normal cells (HUVEC) than the known FGFR inhibitor, BGJ-398 (Figure 1D). The IC_50_ values of MPT0L145 in RT-112 and HUVEC were 11.1 μM and 0.05 μM, respectively. RT-112 cells reportedly rely on FGFRs for growth and are therefore chosen to confirm the effects of MPT0L145 on FGFR signaling [22, 23]. BGJ-398, a known selective inhibitor of FGFR1 to FGFR3, was included as a reference compound. The data revealed that MPT0L145 exerted inhibitory activity on auto-phosphorylation of FGFR1 and FGFR3 as well as its downstream docking protein, FRS2, in 1 h (Figure 2A). The major downstream pathways of FGFRs are MAPK, PI3K/AKT, and PLC-γ. RT-112 cells, which express FGFR3-TACC3, are reportedly unable to activate PLCγ due to a deletion of the last exon of FGFR3 [24]. Next, we examined the kinetic effects of MPT0L145 on the signaling pathways downstream of FGFR from 1 to 8 h in RT-112 cells. MPT0L145 inhibited phosphorylation of ERK at 1 h in a concentration-dependent manner (Figure 2B). The compound displayed better potency than BGJ-398 in inhibiting AKT phosphorylation from 1 to 4 h (Figure 2B, 2C). The phosphorylation of ERK and AKT were fully repressed by MPT0L145 at 8 h (Figure 2D). These data support the observed inhibitory effects of MPT0L145 on FGFR signaling pathways in bladder cancer cells.

### Differential gene expression in MPT0L145-treated cells

**Figure 3:**
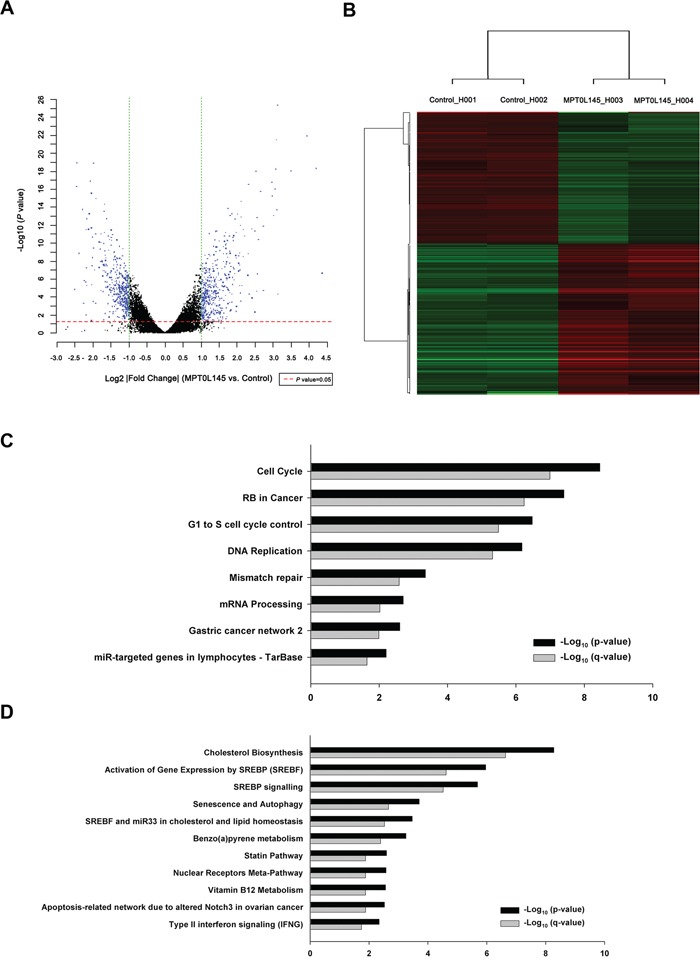
Differentially expressed genes and pathway analysis in MPT0L145-treated cells RT-112 cells were treated with MPT0L145 (4 μM) for 8 h. Total RNA was extracted and subjected to cDNA microarray analysis. **A.** Volcano plot, **B.** Clustering, **C.** Pathway analysis for downregulated genes. **D.** Pathway analysis for upregulated genes.

To further elucidate the mechanisms underlying the anticancer activity of MPT0L145, differential gene expression was analyzed via cDNA microarray. The volcano plot shows the distribution of differentially expressed genes according to fold-change and significance (Figure 3A). The red dotted line represents the *P* value cut-off (0.05), and the green dotted line indicates the fold change cut-off (log2 |fold change|≧ 1). The numbers of upregulated and downregulated genes were 465 and 426, respectively. For advanced data analysis, intensity data were pooled and calculated to identify differentially expressed genes based on the threshold of fold change and *P* value. Correlation of expression profiles between samples and treatment conditions was demonstrated using unsupervised hierarchical clustering analysis (Figure 3B). Gene set enrichment analysis of pathways was performed using differentially expressed gene lists as input and analyzed with the ConsensusPathDB interaction database [25]. The enriched pathways of downregulated (Figure 3C) and upregulated genes (Figure 3D) were plotted on the y-axis versus measure of significance (negative logarithm of the P-value or Q-value) on the x-axis. Among these, genes associated with the pathway of cell cycle progression were decreased (Supplementary Figure S2). Conversely, MPT0L145 increased genes associated with pathway of autophagy and senescence (Supplementary Figure S3). To analyze the possibility of MPT0L145-induced senescence, we examined the activity of β-galactosidase in RT-112 cells. The data showed that there is no appreciable senescence observed in MPT0L145-treated cells, whereas doxorubicin or serum starvation increased β-galactosidase activity (Supplementary Figure S4). To elucidate the mechanisms underlying the anticancer activity of MPT0L145, pathways associated with autophagy and cell cycle progression were selected for further validation.

### MPT0L145 induces cell cycle arrest at the G_0_/G_1_ phase in bladder cancer cells

**Figure 4:**
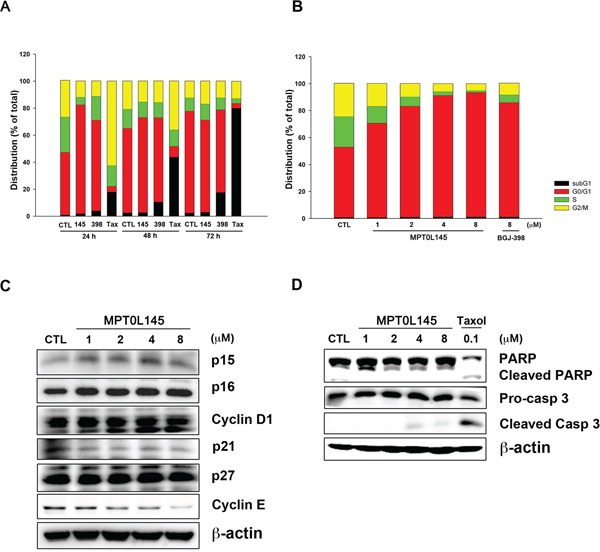
Effects of MPT0L145 on cell cycle distribution **A.** RT-112 cells were treated with MPT0L145 (4 μM), BGJ-398 (4 μM) and Paclitaxel (0.1 μM) for the indicated times, and cell cycle distribution was analyzed via flow cytometry. (CTL: control group, 145: MPT0L145, 398: BGJ-398, Tax: Paclitaxel) **B.** RT-112 cells were exposed to the indicated concentrations of MPT0L145 and BGJ-398 (8 μM) for 24 h and subjected to flow cytometry. **C.** Effects of MPT0L145 on cell cycle regulator proteins. RT-112 cells were treated with different concentrations of MPT0L145 for 24 h and subjected to western blot. **D.** RT-112 cells were treated with MPT0L145 and Paclitaxel for 72 h. Apoptosis was assessed via detection of cleaved caspase-3 and PARP.

In the above experiments, we observed that genes associated with cell cycle progression were decreased in MPT0L145-treated cells. Accordingly, we further examined the effects of MPT0L145 on cell cycle progression in RT-112 cells. The data revealed that MPT0L145 induced G_0_/G_1_ cell cycle arrest to a dramatic extent in 24 h. This phenomenon was also observed in the cells treated with BGJ-398 (Figure 4A). Interestingly, however, MPT0L145 did not promote accumulation of the sub-G_1_ phase, a marker of apoptotic cell death, in 72 h, whereas BGJ-398 and paclitaxel induced pronounced apoptosis at 48 h to 72 h. Moreover, the effect of MPT0L145 on G_0_/G_1_ arrest was concentration-dependent (Figure 4B). These findings indicate that the anti-growth activity of MPT0L145 occurs, at least in part, through disrupting cell cycle progression at the G_0_/G_1_ phase. Next, we examined expression of G_0_/G_1_ regulatory proteins via western blot. Our data showed a slight increase in p16 and marked decrease in cyclin E levels (Figure 4C). The possibility of MPT0L145-induced apoptosis was further eliminated by examining cleavage of caspase-3 and its substrate, PARP, in 72 h, compared with paclitaxel (Figure 4D). The results collectively suggest that MPT0L145 exhibits anti-growth activity in bladder cancer cells, at least partly through inducing G_0_/G_1_ cell cycle arrest.

### Contribution of autophagy to MPT0L145-induced cell death

**Figure 5:**
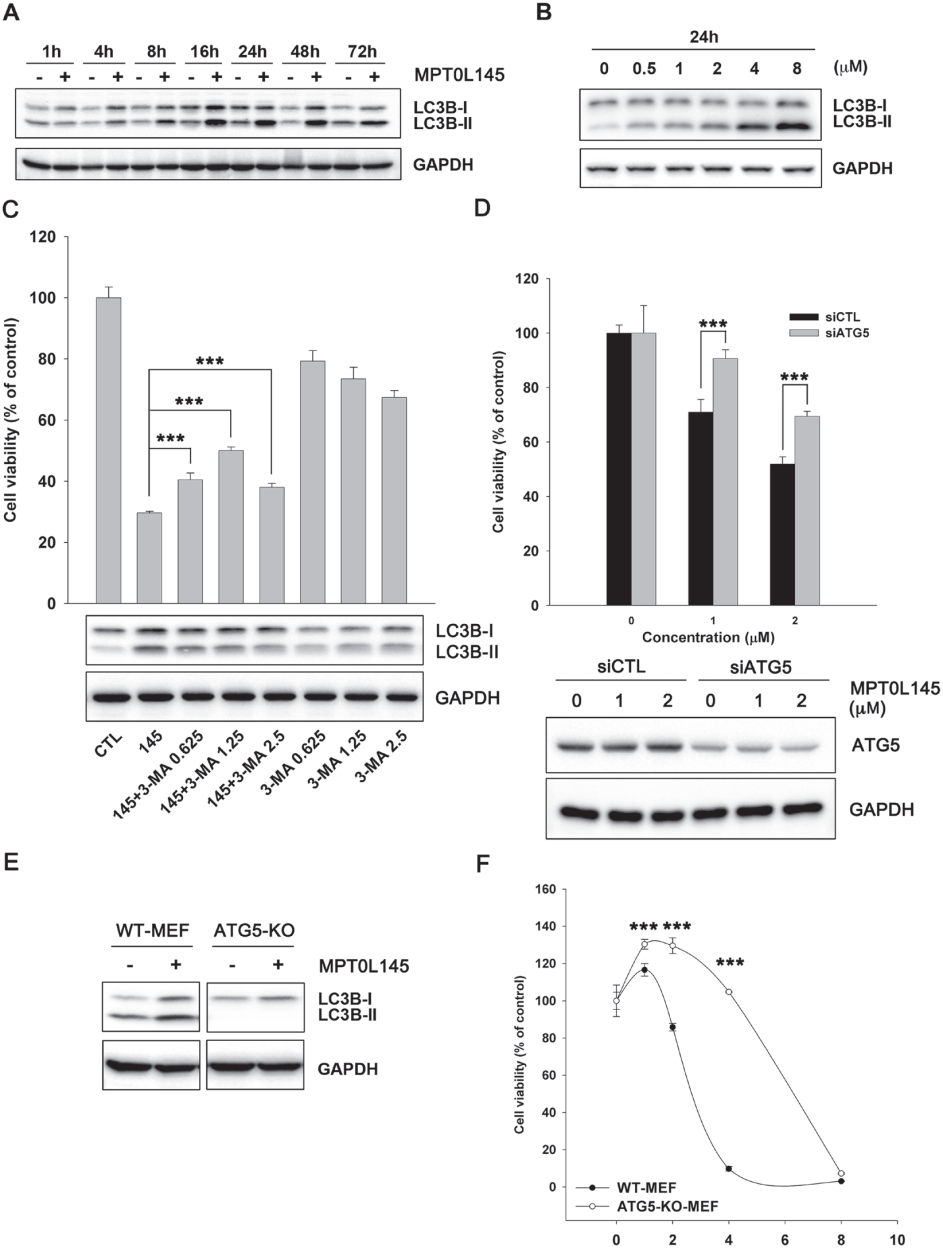
Contribution of autophagy to MPT0L145-induced cell death RT-112 cells were treated with MPT0L145 (4 μM) for the indicated times **A.** or different concentrations for 24 h **B.**, and protein lysates subjected to western blot analysis. **C.** RT-112 cells were treated with MPT0L145 (4 μM) in the presence or absence of autophagy inhibitor, 3-methyladenine (3-MA, 0.625~2.5 mM). Cell viability was assessed with the MTT assay (upper panel). Data are expressed as means ± S.D. (***P < 0.001 compare to MPT0L145 alone). Protein lysates were subjected to western blot analysis (lower panel). **D.** RT-112 cells were transiently transfected with indicated siRNA for 24 h and then treated with MPT0L145 for 72 h. Cell viability was assessed with the MTT assay (upper panel). Data are expressed as means ± S.D. (***P < 0.001, siCTL v.s. siATG5). Protein lysates were subjected to western blot analysis to show the knockdown efficiency (lower panel). **E.** Wild-type (WT) MEF and ATG5 knockout (KO) MEF were treated with MPT0L145 (4 μM) for 24 h, and protein lysates subjected to western blot analysis. **F.** WT MEF and ATG5-KO-MEF were treated with different concentrations of MPT0L145 for 72 h. Cell viability was assessed with the MTT assay. Data are expressed as means ± S.D. (***P < 0.001, WT-MEF v.s. ATG5-KO-MEF).

Our experiments clearly revealed upregulation of genes associated with autophagy by MPT0L145 (Figure 3D). Based on reports that FGFR is involved in suppression of autophagy [26, 27], we hypothesized that MPT0L145 induces autophagy in bladder cancer cells. MPT0L145 induced the conversion of LC3B-I to LC3B-II in a time- and concentration-dependent manner (Figure 5A and 5B). The role of autophagy in MPT0L145-induced cell death was further clarified in RT-112 cells. The data showed that drug-related cytotoxic effects and the conversion of LC3B-I to LC3B-II were reversed by autophagy inhibitor, 3-MA, in a concentration-dependent manner (Figure 5C). Moreover, knocking down of ATG5 also showed rescuing effects on cell viability (Figure 5D). Accordingly, we further examined the effect of MPT0L145 in ATG5-knockout (KO) mouse embryonic fibroblast (MEF). Autophagy was activated in wild-type MEF treated with MPT0L145, but this phenomenon was totally abrogated in ATG5-KO MEF (Figure 5E). We observed reversal of effects on cell viability upon depletion of ATG5 (Figure 5F). The induction of autophagy was also observed in MPT0L145-sensitive cell lines (KMS11, RT4), but not in HUVEC cells (Supplementary Figure 5). The results further support the negative correlation between autophagy and cell viability upon the treatment of MPT0L145. Collectively, our data provide evidence of the contribution of autophagy to MPT0L145-induced cell death.

### Antitumor activity of MPT0L145 in RT-112 xenograft model

**Figure 6:**
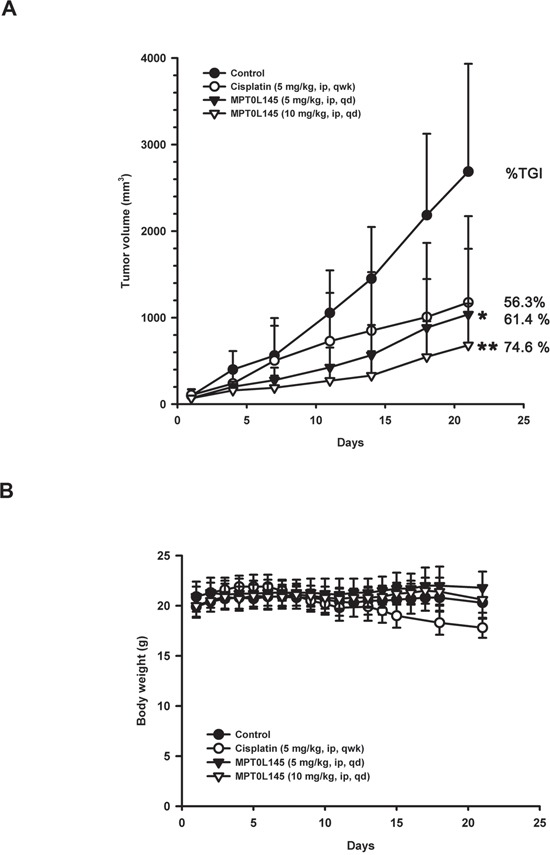
*In vivo* antitumor activity of MPT0L145 in the RT-112 xenograft model Athymic nude mice bearing subcutaneously established RT-112 xenograft tumors were randomized to four groups (n=5) received the indicated treatments via intraperitoneal injection (ip). **A.** Tumor growth volume curves were expressed as means ± S.D., and the percentage of TGI determined. (*P < 0.05, **P < 0.01 compared with the control group) **B.** Body weights were measured, and are expressed as means ± S.D.

To evaluate the anticancer activity of MPT0L145 in the preclinical setting, we examined its effects in athymic nude mice bearing established RT-112 tumor xenografts. MPT0L145 significantly suppressed tumor growth in a dose-dependent manner (Figure 6A). The percentages of tumor growth inhibition (% TGI) of cisplatin (5 mg/kg) and MPT0L145 (5 and 10 mg/kg) are 56.3%, 61.4% and 74.6%, respectively. MPT0L145 exhibited not only comparable antitumor activity to cisplatin, but also better safety, as established from assessment of body weight loss after treatment (Figure 6B). Accordingly, we conclude that MPT0L145 possesses significant antitumor activity *in vivo*.

## DISCUSSION

In the present study, we provided preclinical evidence supporting anticancer activity of the novel selective pan-FGFR inhibitor, MPT0L145. Data from the *in vitro* kinase activity assay showed that MPT0L145 is a potent inhibitor of FGFR1, FGFR2, and FGFR3, and exhibits selectivity against other receptor tyrosine kinases, including EGFR, Erbb2, IGF1R, KIT, FLT3 and VEGFR2 (Supplementary Table S2). From a panel of 16 cells, MPT0L145 activity was more potent in cells with aberrant FGFR activation, along with lower toxicity to normal cells (Supplementary Table S3). The compound inhibited auto-phosphorylation of FGFR1 and FGFR3 as well as components of their downstream signaling pathways, pFRS2, pERK, and pAKT. MPT0L145 exerted anti-growth activity, in part, through inducing G_0_/G_1_ cell cycle arrest. Interestingly, our data suggest that induction of autophagic reaction contributes to MPT0L145-induced cell death. Finally, MPT0L145 exhibited a comparable antitumor activity to cisplatin with better safety in a RT-112 xenograft model *in vivo*. These findings collectively support the potential utility of MPT0L145 as a therapeutic agent for the patients with FGFR-activated bladder cancers.

Deregulation of the FGF/FGFR pathway has been implicated in a variety of human malignancies and cancer progression, providing a strong rationale for the development of anti-FGF/FGFR therapeutic agents. Currently, the most clinically advanced FGFR inhibitors are non-selective TKIs, such as nintedanib, lenvatinib, ponatinib, and dovitinib, with multiple targets, such as VEGFRs, PDGFRs KIT, and FLT3 [28–31]. Recently efforts have focused on the development of selective FGFR inhibitors or monoclonal antibodies, such as AZD-4547, BGJ-398, LY2874455, and MGFR1877S [12]. For instance, BLU9931, an irreversible inhibitor of FGFR4, has been developed as targeted therapy for patients with FGFR4-actvated hepatocellular carcinoma [32]. The toxicity profiles of non-selective and selective FGFR inhibitors are different. The dose-limiting toxicities due to inhibition of FGFR1/FGFR3 and FGFR4 are hyperphosphatemia and the blockade of bile acid synthesis, respectively [33–35]. In addition, toxicities of non-selective FGFR inhibitors, such as hypertension, cardiovascular events and proteinuria, are induced from the inhibition of VEGFR [36]. In the present study, MPT0L145 exerts significantly lower toxicity to HUVECs than BGJ-398, possibly resulting from loss of anti-VEGFR activity.

Bladder cancer is estimated as the fourth most common cancer in men and the eighth leading cause of cancer-related death in the United States in 2015 [14]. Early-stage cancers may be removed by surgery, followed by administration of intravesical BCG (bacillus Calmette-Guérin) therapy or chemotherapeutic drugs. Advanced cancers may require cystectomy, and improved outcomes have been reported with the use of chemotherapy. So far, the most active chemotherapy regimens for MIBC and metastatic bladder carcinoma are MVAC (methotrexate, vinblastine, doxorubicin, and cisplatin), dose-dense MVAC, and gemcitabine plus cisplatin [37, 38]. However, no molecular targeted therapies have been approved for treatment of this disease as yet. Recently, FGFR family gene fusions have been reported as emerging therapeutic targets for a wide spectrum of solid tumors, including bladder cancer [18]. Comprehensive molecular characterization of urothelial bladder carcinoma by The Cancer Genome Atlas (TCGA) led to the identification of FGFR3-TACC3 fusions as recurrent in-frame activating translocations [39]. FGFR3-TACC3 fusions are reported to exist as dimers in the absence of growth factor stimulation, leading to constitutively activation of FGFR3 [40]. Interestingly, cancer cells expressing FGFR3-TACC3 in this study (RT-112, RT4, HepG2) were highly sensitive to MPT0L145 (Supplementary Table S3). The results suggest that genomic examination of FGFR3-TACC3 may provide criteria to identify optimal responders in the clinic in the future.

Autophagy is a catabolic process by which a cell “eats” itself and leads to cell death or paradoxically allows cells to escape death induced by chemotherapy or radiation [41]. Receptor tyrosine kinase inhibitors, such as EGFR inhibitors, have been reported to induce a cytoprotective response in lung cancer cells [42]. Here, we demonstrated that MPT0L145 induces autophagy in bladder cancer cells (Figure 5A and 5B). MPT0L145-induced cytotoxicity was rescued by autophagy inhibitor and knocking down of ATG5 (Figure 5C and 5D). In mouse embryonic fibroblast (MEF), which reportedly expresses functional FGFR1 at the cell surface [43], both autophagic response and cytotoxic effects were reversed upon elimination of ATG5 (Figure 5E and 5F). Moreover, the observation was further supported by the negative correlation between cell viability and the induction of autophagy (Supplementary Figure 5A). The data also showed that another selective FGFR inhibitor, BGJ-398, increased the expression of LC3B-II in a time-dependent manner (Supplementary Figure 5B). To our knowledge, the current findings have demonstrated for the first time that FGFR inhibitor induces cytotoxic autophagy in cancer cells.

In conclusion, our findings provide compelling evidence of the significant anti-tumor activity of MPT0L145 in a preclinical model, supporting its potential as a potentially safe and effective therapeutic agent for FGFR-dysregulated cancers.

## MATERIALS AND METHODS

### Synthesis of compound 1 (MPT0L145) and compounds 23-35

Synthetic schemes, detailed procedures can be found in Figure 1, Supplementary Figure S1 and the Supplementary Methods. Compounds 1 (MPT0L145) and 23-35 mentioned in this manuscript are covered by US provisional patent application 62/194,705 filed on July 20, 2015.

### Cell lines, antibodies, and reagents

KMS-11, the human myeloma cell line displacing the t(4;14) translocation and overexpression of FGFR3, was cultured as previously described [44]. Human sarcoma cell lines (FUDDLS, LISA-2) were cultured as our previous study [45]. Human umbilical vein endothelial cells (HUVEC) was purchased and cultured as suggested methods from Bioresource Collection and Research Center (BCRC; Hsinchu, Taiwan). Other mentioned cell lines were purchased from American Type Culture Collection (Manassas, VA, USA) and cultured with suggested media with 10% fetal bovine serum (FBS) and 1% penicillin-streptomycin (GIBCO, Grand Island, NY, USA) at 37°C in a humidified incubator containing 5% CO_2_. 3-(4,5-Dimethylthiazol-2-yl)-2,5-diphenyltetrazolium bromide (MTT), propidium iodide (PI) and all of the other chemical reagents were purchased from Sigma Chemical (St. Louis, MO, USA). BGJ398 was purchase from Sellekchem (Houston, TX). Antibodies against various proteins were obtained from the following sources: PARP (Poly-ADP-ribose polymerase), p15, p16, cyclin D1, p21, p27 and cyclin E were obtained from Santa Cruz Biotechnology Inc. (Santa Cruz, CA, USA). Caspase-3, AKT, pAKT (Ser473), ERK, pERK (Thr202/Tyr204), pFGFR1 (Tyr635/654) and pFRS2 (Tyr 436) were obtained from Cell Signaling Technology (Danvers, MA, USA). FGFR1, FGFR3, FRS2, LC3-B, GAPDH and β-actin were purchased from Genetex (Irvine, CA, USA). pFGFR3 (Tyr 724) was purchased from Abcam (Cambridge, MA, USA).

### Cell viability assay

Cells were seeded in 96-well plates and exposed to DMSO, or indicated compounds for 72 hours. Cell viability was assessed using the 3-(4,5-dimethylthiazol-2-yl)-2,5-diphenyltetrazolium bromide (MTT) assay as described previously [46]. Briefly, 100 μl of 0.5 mg/ml MTT were added to each well and incubated at 37°C for 1 hour. After that, 100 μl of extraction reagents (0.1 M sodium acetate buffer for suspension cells or DMSO for attached cells) were added to each well to lyse cells and absorbance at 550 nm was measured. Cell viability was expressed as the percentage of surviving cells in drug-treated versus DMSO-treated control cells (which was considered as 100% viability). The concentration that inhibits 50% of cell growth (IC_50_) were determined according to the dose-effect curves in Figure 1C, Figure 1D and Supplementary Figure S6.

### Kinome profiling of MPT0L145

The inhibitory activities of MPT0L145 on protein kinases were assessed by the service of KINOMEscan^®^ (DiscoverRx, Fremont, CA, USA). The screening is based on a competition-binding assay and the *K*_d_ values were determined as described previously [47]. The dose-effective curves of those kinases with Kd values lower than 10,000 nM were plotted in Supplementary Figure S7.

### Flow cytometric analysis

Cells were seeded in 6-well plates and treated with DMSO or indicated compounds at various concentrations for the indicated times. After treatment, the cells were harvested by trypsinization, washed with PBS, and the pellets were fixed in ice-cold 70% ethanol at -20°C overnight. After centrifugation, the cells were incubated for 15 minutes at room temperature in 0.1 mL of phosphate-citric acid buffer (0.2 M NaHPO4, 0.1 M citric acid, and pH 7.8) and then stained with propidium iodide staining buffer containing Triton X-100 (0.1%, v/v), RNase A (100 μg/mL), and propidium iodide (80 μg/mL) for 30 minutes in the dark. DNA contents were analyzed by flow cytometry with CellQuest software (Becton Dickinson, Mountain View, CA, USA). The results were obtained from three independent experiments and the representative data were shown in Supplementary Figure S8 and S9.

### Immunoblot analysis and small interfering RNA transfection

Cells were seeded and treated with indicated compounds at various concentrations for the indicated times. After treatment, cells were harvested by scraping with RIPA buffer containing protease inhibitors and phosphatase inhibitors (Roche Diagnostics Corp., Indianapolis, IN, USA). Total cell lysates were centrifuged at 13,000 g for 30 min and equal amounts of protein were separated by SDS–PAGE and immunoblotted with specific antibodies as previous described [46]. The cells were allowed to recover for 24 h before following treatment. The small interfering RNA (siRNA) for Atg5 (HSS114103, HSS114104, HSS190366), and negative control (siCTL) were purchased from Invitrogen (Carlsbad, CA, USA). Transient transfection was performed by using lipofectamine 2000 according to the manufacture's instructions.

### RNA isolation and microarray analysis

RT-112 cells were treated with MPT0L145 for 8 h, and total RNA were isolated by RNeasy Mini Kit according to instructions from the manufacture (Qiagen, Valencia, CA, USA). RNA quality was determined by 2100 Bioanalyzer (Agilent Technologies, Santa Clara, CA, USA). DNA-free total RNA were used to probe human OneArray (Phalanx Biotech Group) to detect differentially expressed genes. GPR files were loaded into Rosetta Resolver^®^ System (Rosetta Biosoftware) to process data analysis. Standard selection criteria to identify differentially expressed genes are log2 |Fold change| ≥ 1 and *P* values < 0.05. Gene clustering by averagely linkage algorithm performed on selected differentially expressed gene lists after data transformation and mean centering. Pathway analysis was performed using differentially expressed gene lists as input and analyzed with the ConsensusPathDB interaction database [25] and WikiPathways [48]. Differentially expressed genes were highlighted in the pathway by PathVisio [49]. The gene lists and the results of pathway analysis were shown in Supplementary Files 1 and 2.

### SA-β-galactosidase staining

Cells were seeded in 6-well plates and treated with indicated agents for 24h. Senescence was measured by Senescence β-Galactosidase Staining Kit according to the manufacture's instruction (Cell Signaling Technology, Danvers, MA, USA).

### *In vivo* xenograft model

Eight-week-old female athymic nude mice were group-housed in the TMU Laboratory Animal Center (Taipei, Taiwan) under conditions of constant photoperiod (12 h light/12 h dark at 21–0023°C and 60–85% humidity) with ad libitum access to sterilized food and water. All animal experiments followed ethical standards, and protocols as previously described [50]. Each mouse was inoculated subcutaneously with 1×10^6^ RT-112 cells in a total volume of 0.1 mL serum-free medium containing 50% Matrigel (BD Biosciences). As tumors became established (~100 mm^3^), mice were randomized to four groups (n = 5) that received the following treatments: (a) 0.5% carboxymethyl cellulose/0.1%Tween 80 vehicle, (b) cisplatin at 5 mg/kg/wk, MPT0L145 at (c) 5 mg/kg/d or (d) 10 mg/kg/d by intraperitoneal injection (ip). Tumors were measured weekly using calipers. Tumor volume (mm^3^) was calculated from w^2^×l/2 (w = width, l= length in mm of the tumor).

### Statistical analysis

Each experiment was performed at least three times. Data in bar graph are given as the means ± S.D. Means were checked for statistical difference using the t-test and *P* values less than 0.05 were considered significant (*P < 0.05, **P < 0.01, ***P < 0.001).

## SUPPLEMENTARY MATERIAL AND METHODS FIGURES AND TABLES






