# Supplementary material for: Trichlorobenzene-substituted azaaryl compounds as novel FGFR inhibitors exhibiting potent antitumor activity in bladder cancer cells in vitro and in vivo
Source: Oncotarget. 2016 Mar 25;7(18):26374–87. doi: 10.18632/oncotarget.8380 (PMC5041986; doi:10.18632/oncotarget.8380)
Supplement: Supplementary file 1 [file oncotarget-07-26374-s001.pdf]

SUPPLEMENTARY MATERIAL AND METHODS

Synthesis of compound 1 (MPT0L145)

2,4-dichloro-1,3,5-triazine (b)

A mixture of sodium dicyanamide (**a**) (3.0g, 33.70mmol) was dissolved in H_2O (13ml) and added to another flask which filled with conc. HCl (15ml) at -78°C. The reaction stirred at -78°C for 15min then heated to -35°C for 15min. Let the reaction cool to the 0°C and filtered to get the precipitant. Take another flask filled with DM (10ml) at room temperature and added the $POCl_3$ (1.84ml, 19.71mmol) and DMF (1.53ml, 19.71mmol) at 0°C. After stirred a while, the reaction was added the above precipitant portion-wisely and stirred at room temperature for overnight. The reaction was quenched by water and extracted by ethyl acetate (30ml x 3). The residue was purified by flash column over silica gel (ethyl acetate: n-Hexane = 1:4, Rf = 0.63) to afford **b** (0.64g, 13.13%) as a white solid. ^{1}H-NMR (300MHz, $CDCl_3$): δ 8.90 (s, 1H).

4-chloro-*N*-methyl-1,3,5-triazin-2-amine (c)

A mixture of **b** (0.10g, 0.67mmol) and IPA (3ml) was stirred for a while then added the 2M methylamine in THF (0.67ml, 1.34mmol) at 0°C and stirred back to room temperature for overnight. The reaction was quenched by water and extracted by ethyl acetate (30ml x 3). The residue was purified by flash column over silica gel (ethyl acetate: n-Hexane = 1:1, Rf= 0.50) to afford **c** (0.07g, 72.27%) as a pale white solid. ^{1}H-NMR (300MHz, $CDCl_3$): δ 3.03-3.06 (m, 3H), 5.71 (br, 1H), 8.36 (d, *J*= 42.0 Hz, 1H).

N^2-(4-(4-ethylpiperazin-1-yl)phenyl)-*N*^{4}-methyl-1,3,5-triazine-2,4-diamine (d)

A mixture of 4-(4-Ethyl-piperazin-1-yl)-phenylamine (0.10g, 0.48mmol), AcOH (2ml) and H_2O (0.5ml) was added the **c** (0.07g, 0.48mmol) and refluxed for overnight. The reaction was quenched by saturated $NaHCO_3$ (aq.) and extracted by ethyl acetate (30ml x 3). The residue was purified by flash column over silica gel (dichloromethane: methanol = 9:1, Rf = 0.28) to afford **d** (0.05g, 33.24%) as a pale yellow solid. ^{1}H-NMR (300MHz, $CDCl_3$): δ 1.13 (t, *J*= 7.2 Hz, 3H), 2.46 (q, *J*= 7.2 Hz, 2H), 2.62 (t, *J*= 5.1 Hz, 4H), 2.98 (d, *J*= 5.1 Hz, 3H), 3.19 (t, *J*= 5.1 Hz, 4H), 5.17-5.29 (m, 1H), 5.55 (br, 1H), 6.91 (d, *J*= 8.7 Hz, 2H), 7.37 (d, *J*= 8.1 Hz, 1H), 7.46 (s, 1H), 8.20 (d, *J*= 44.7 Hz, 1H).

1-(4-((4-(4-ethylpiperazin-1-yl)phenyl)amino)-1,3,5-triazin-2-yl)-1-methyl-3-(2,4,6-trichloro-3,5-dimethoxyphenyl)urea (1)

A mixture of 2,4,6-Trichloro-3,5-dimethoxy-phenylamine (0.39g, 1.52mmol) and *p*-dioxane (7ml) was added the triphosgene (0.75g, 2.52mmol) dissolved in toluene (3ml) and then stirred and refluxed for overnight. The reaction was directly removed out solvent and dissolved in toluene (10ml). The mixture was added the **d** (0.40g, 1.28mmol) and stirred and refluxed for overnight. The reaction was quenched by saturated $NaHCO_3$(aq.) and extracted by ethyl acetate (30ml x 3). The residue was purified by flash column over silica gel (dichloromethane: methanol = 15:1, Rf = 0.35) to afford **1** (0.12g, 15.73%) as a pale yellow solid. ^{1}H-NMR (500MHz, $CDCl_3$+CD_3OD): δ 1.02 (t, *J*= 7.5 Hz, 3H), 2.37 (q, *J*= 7.5 Hz, 2H), 2.46-2.53 (m, 4H), 2.89-3.09 (m, 4H), 3.41 (s, 3H), 3.76 (s, 3H), 3.79 (s, 3H), 6.59-6.84 (m, 2H), 7.14-7.31 (m, 2H), 8.32 (s, 1H).

Synthesis of compound 23-35

6-chloro-*N*-methylpyrimidin-4-amine (8)

A mixture of 4,6-dichloropyrimide (0.50g, 3.36mmol) and IPA (1.5ml) was stirred for a while then added the 2M methylamine in THF (4.2ml, 8.40mmol) at 0°C and stirred back to room temperature for overnight. The reaction was quenched by water and extracted by ethyl acetate (30ml x 3). The residue was purified by flash column over silica gel (ethyl acetate: n-Hexane = 1:2, Rf= 0.20) to afford **8** (0.47g, 97.43%) as a pale yellow solid. ^{1}H-NMR (300MHz, $CDCl_3$): δ 2.95 (d, *J*= 5.1 Hz, 3H), 5.26 (br, 1H), 6.34 (s, 1H), 8.34 (s, 1H).

6-chloro-*N*-methyl-2-phenylpyrimidin-4-amine (9)

A mixture of 2-phenyl-4,6-dichloropyrimidine (0.25g, 1.11mmol) and IPA (3ml) was stirred for a while then added the 2M methylamine in THF (1.39ml, 2.78mmol) at 0°C and stirred back to room temperature for overnight. The reaction was quenched by water and extracted by ethyl acetate (30ml x 3). The residue was purified by flash column over silica gel (ethyl acetate: n-Hexane = 1:4, Rf= 0.18) to afford **9** (0.22g, 90.23%) as a pale yellow solid. ^{1}H-NMR (300MHz, $CDCl_3$): δ 3.02 (d, *J*= 4.2 Hz, 3H), 5.09 (br, 1H), 6.26 (s, 1H), 7.42-7.46 (m, 3H), 8.35-8.37 (m, 2H).

N[4]-(4-(4-ethylpiperazin-1-yl)phenyl)-*N*[6]-methylpyrimidine-4,6-diamine (10)

A mixture of 4-(4-Ethyl-piperazin-1-yl)-phenylamine (0.49g, 2.39mmol), H_2O (0.75ml) and AcOH (3ml) was added the **8** (0.15g, 3.13mmol) and refluxed for overnight. The reaction was quenched by saturated $NaHCO_3$ (aq.) and extracted by ethyl acetate (30ml x 3). The residue was purified by flash column over silica gel (dichloromethane: methanol = 9:1, Rf = 0.25) to afford **10** (0.60g, 80.20%) as a pale orange solid. ^{1}H-NMR (300MHz, $CDCl_3$): δ 1.16 (t, *J*= 7.2 Hz, 3H), 2.48 (q, *J*= 7.2 Hz, 2H), 2.64 (t, *J*= 5.1 Hz, 4H), 2.81 (d, *J*= 5.4 Hz, 3H), 3.22 (t, *J*= 5.1 Hz, 4H), 4.86 (s, 1H), 5.54 (s, 1H), 6.69 (s, 1H), 6.95 (d, *J*= 9.0 Hz, 2H), 7.18 (d, *J*= 8.7 Hz, 2H), 8.14 (s, 1H).

N[4]-(4-(4-ethylpiperazin-1-yl)phenyl)-*N*[6]-methyl-2-phenylpyrimidine-4,6-diamine (11)

A mixture of 4-(4-Ethyl-piperazin-1-yl)-phenylamine (0.16g, 0.77mmol), H_2O (0.4ml) and AcOH (1.6ml) was added the **9** (0.22g, 1.00mmol) and refluxed for overnight. The reaction was quenched by saturated $NaHCO_3$ (aq.) and extracted by ethyl acetate (30ml x 3). The residue was purified by flash column over silica gel (dichloromethane: methanol = 9:1, Rf = 0.35) to afford **11** (0.22g, 73.54%) as a pale yellow solid. ^{1}H-NMR (500MHz, $CDCl_3$): δ 1.16 (t, *J*= 7.5 Hz, 3H), 2.52 (q, *J*= 7.5 Hz, 2H), 2.66 (t, *J*= 5.0 Hz, 4H), 2.88 (d, *J*= 5.0 Hz, 3H), 3.25 (t, *J*= 5.0 Hz, 4H), 4.84 (s, 1H), 5.51 (s, 1H), 6.51 (s, 1H), 6.95 (d, *J*= 8.5 Hz, 2H), 7.23 (d, *J*= 8.5 Hz, 2H), 7.42-7.43 (m, 3H), 8.31-8.33 (m, 2H).

6-(4-(4-ethylpiperazin-1-yl)phenyl)-N-methylpyrimidin-4-amine (12)

A mixture of 1-(4-bromophenyl)piperazine (0.15g, 0.62mmol) and acetone (5ml) was added the potassium carbonate (0.17g, 1.24mmol) and ethyl iodide (0.08ml, 1.00mmol) and stirred at room temperature for 4hrs. The residue was purified by flash column over silica gel (dichloromethane: methanol = 9:1, Rf = 0.43) to get the product. A mixture of above product and p-dioxane (10ml) was added the $PdCl_2$dppf (0.04g, 0.06mmol), potassium acetate (0.55g, 5.58mmol) and bispinacolactoboron (0.71g, 2.79mmol) and stirred and refluxed for overnight. The residue was purified by flash column over silica gel (dichloromethane: methanol = 9:1, Rf = 0.48) to get the product. A mixture of **8** (0.06g, 0.41mmol), H_2O (1ml) and p-dioxane (4ml) was added the $PdCl_2$dppf (0.03g, 0.04mmol), cesium carbonate (0.27g, 0.82mmol) and above product (0.13g, 0.41mmol) then stirred and refluxed for overnight. The reaction was quenched by saturated $NaHCO_3$(aq.) and extracted by ethyl acetate (30ml x 3). The residue was purified by flash column over silica gel (dichloromethane: methanol = 19:1, Rf = 0.19) to afford **12** (0.08g, 65.61%) as a pale yellow solid. ^{1}H-NMR (500MHz, $CDCl_3$): δ 1.16 (t, *J*= 7.0 Hz, 3H), 2.52 (d, *J*= 7.0 Hz, 2H), 2.65 (s, 4H), 3.00 (d, *J*= 5.0 Hz, 3H), 3.35 (s, 4H), 4.92 (s, 1H), 6.62 (s, 1H), 6.97 (d, *J*= 7.0 Hz, 2H), 7.93 (d, *J*= 9.0 Hz, 2H), 8.59 (s, 1H).

N-methyl-6-phenylpyrimidin-4-amine (13)

A mixture of **8** (0.28g, 1.95mmol), H_2O (1.5ml) and p-dioxane (6ml) was added the $PdCl_2$dppf (0.14g, 0.20mmol), cesium carbonate (1.27g, 3.90mmol) and phenylboronic acid (0.24g, 1.95mmol) then stirred and refluxed for overnight. The reaction was quenched by saturated $NaHCO_3$(aq.) and extracted by ethyl acetate (30ml x 3). The residue was purified by flash column over silica gel (ethyl acetate: n-Hexane = 1:2, Rf = 0.30) to afford **13** (0.25g, 69.21%) as a pale yellow solid. ^{1}H-NMR (300MHz, CD_3OD): δ 2.95 (s, 3H), 6.84 (s, 1H), 7.45-7.48 (m, 3H), 7.89 (s, 2H), 8.45 (s, 1H).

6-(4-methoxyphenyl)-*N*-methylpyrimidin-4-amine (14)

A mixture of **8** (0.28g, 1.95mmol), H_2O (1.5ml) and p-dioxane (6ml) was added the $PdCl_2$dppf (0.14g, 0.20mmol), cesium carbonate (1.27g, 3.90mmol) and 4-methoxybenzeneboronic acid (0.30g, 1.95mmol) then stirred and refluxed for overnight. The reaction was quenched by saturated $NaHCO_3$(aq.) and extracted by ethyl acetate (30ml x 3). The residue was purified by flash column over silica gel (ethyl acetate: n-Hexane = 1:1, Rf = 0.18) to afford **14** (0.30g, 71.47%) as a pale yellow solid. ^{1}H-NMR (300MHz, $CDCl_3$): δ 3.01 (d, *J*= 5.1 Hz, 3H), 3.86 (s, 3H), 5.04 (s, 1H), 6.64 (s, 1H), 6.98 (d, *J*= 9.0 Hz, 2H), 7.96 (d, *J*= 8.7 Hz, 2H), 8.61 (s, 1H).

6-(4-fluorophenyl)-*N*-methylpyrimidin-4-amine (15)

A mixture of **8** (0.28g, 1.95mmol), H_2O (1.5ml) and p-dioxane (6ml) was added the $PdCl_2$dppf (0.14g, 0.20mmol), cesium carbonate (1.27g, 3.90mmol) and 4-fluorobenzeneboronic acid (0.27g, 1.95mmol) then stirred and refluxed for overnight. The reaction was quenched by saturated $NaHCO_3$(aq.) and extracted by ethyl acetate (30ml x 3). The residue was purified by flash column over silica gel (ethyl acetate: n-Hexane = 1:2, Rf = 0.15) to afford **15** (0.16g, 40.38%) as a pale yellow solid. ^{1}H-NMR (300MHz, $CDCl_3$): δ 3.02 (d, *J*= 5.1 Hz, 3H),

5.10 (s, 1H), 6.65 (s, 1H), 7.12-7.18 (m, 2H), 7.96-8.01 (m, 2H), 8.63 (s, 1H).

6-(4-chlorophenyl)-*N*-methylpyrimidin-4-amine (16)

A mixture of **8** (0.28g, 1.95mmol), H_2O (1.5ml) and p-dioxane (6ml) was added the $PdCl_2dppf$ (0.14g, 0.20mmol), cesium carbonate (1.27g, 3.90mmol) and 4-chlorobenzeneboronic acid (0.30g, 1.95mmol) then stirred and refluxed for overnight. The reaction was quenched by saturated $NaHCO_3$(aq.) and extracted by ethyl acetate (30ml x 3). The residue was purified by flash column over silica gel (ethyl acetate: n-Hexane = 1:2, Rf = 0.06) to afford **16** (0.20g, 46.69%) as a pale yellow solid. ^{1}H-NMR (300MHz, $CDCl_3$): δ 3.02 (d, *J*= 5.1 Hz, 3H), 5.11 (s, 1H), 6.66 (s, 1H), 7.44 (d, *J*= 8.7 Hz, 2H), 7.93 (d, *J*= 8.7 Hz, 2H), 8.63 (s, 1H).

4-(6-(methylamino)pyrimidin-4-yl) benzonitrile (17)

A mixture of **8** (0.28g, 1.95mmol), H_2O (1.5ml) and p-dioxane (6ml) was added the $PdCl_2dppf$ (0.14g, 0.20mmol), cesium carbonate (1.27g, 3.90mmol) and 4-cyanobenzeneboronic acid (0.29g, 1.95mmol) then stirred and refluxed for overnight. The reaction was quenched by saturated $NaHCO_3$(aq.) and extracted by ethyl acetate (30ml x 3). The residue was purified by flash column over silica gel (ethyl acetate: n-Hexane = 1:2, Rf = 0.23) to afford **17** (0.30g, 73.18%) as a pale yellow solid. ^{1}H-NMR (300MHz, CD_3OD): δ 3.05 (d, *J*= 5.4 Hz, 3H), 5.21 (s, 1H), 6.73 (s, 1H), 7.76 (d, *J*= 8.7 Hz, 2H), 8.10 (d, *J*= 8.7 Hz, 2H), 8.67 (s, 1H).

3-(6-(methylamino)pyrimidin-4-yl) benzonitrile (18)

A mixture of **8** (0.28g, 1.95mmol), H_2O (1.5ml) and p-dioxane (6ml) was added the $PdCl_2dppf$ (0.14g, 0.20mmol), cesium carbonate (1.27g, 3.90mmol) and 3-cyanobenzeneboronic acid (0.29g, 1.95mmol) then stirred and refluxed for overnight. The reaction was quenched by saturated $NaHCO_3$(aq.) and extracted by ethyl acetate (30ml x 3). The residue was purified by flash column over silica gel (ethyl acetate: n-Hexane = 1:1, Rf = 0.13) to afford **18** (0.33g, 80.45%) as a pale yellow solid. ^{1}H-NMR (300MHz, CD_3OD): δ 2.97 (s, 3H), 6.93 (s, 1H), 7.67 (t, *J*= 7.8 Hz, 1H), 7.83 (d, *J*= 7.8 Hz, 1H), 8.23 (d, *J*= 8.7 Hz, 1H), 8.30 (s, 1H), 8.49 (s, 1H).

N-methyl-6-(3-nitrophenyl)pyrimidin-4-amine (19)

A mixture of **8** (0.28g, 1.95mmol), H_2O (1.5ml) and p-dioxane (6ml) was added the $PdCl_2dppf$ (0.14g, 0.20mmol), cesium carbonate (1.27g, 3.90mmol) and 3-nitrobenzeneboronic acid (0.33g, 1.95mmol) then stirred and refluxed for overnight. The reaction was quenched by saturated $NaHCO_3$(aq.) and extracted by ethyl acetate (30ml x 3). The residue was purified by flash column over silica gel (ethyl acetate: n-Hexane = 1:1, Rf = 0.38) to afford **19** (0.19g, 42.32%) as a pale yellow solid. ^{1}H-NMR (300MHz, CD_3OD): δ 2.98 (s, 3H), 6.98 (s, 1H), 7.74 (t, *J*= 8.1 Hz, 1H), 8.34 (d, *J*= 8.1 Hz, 2H), 8.51 (s, 1H), 8.80 (s, 1H).

6-(furan-2-yl)-*N*-methylpyrimidin-4-amine (20)

A mixture of **8** (0.28g, 1.95mmol), H_2O (1.5ml) and p-dioxane (6ml) was added the $PdCl_2dppf$ (0.14g, 0.20mmol), cesium carbonate (1.27g, 3.90mmol) and 2-furanboronic acid (0.22g, 1.95mmol) then stirred and refluxed for overnight. The reaction was quenched by saturated $NaHCO_3$(aq.) and extracted by ethyl acetate (30ml x 3). The residue was purified by flash column over silica gel (ethyl acetate: n-Hexane = 2:1, Rf = 0.13) to afford **20** (0.10g, 29.27%) as a pale yellow solid. ^{1}H-NMR (300MHz, CD_3OD): δ 3.01 (d, *J*= 5.1 Hz, 3H), 5.11 (s, 1H), 6.55 (d, *J*= 5.1 Hz, 1H), 6.68 (s, 1H), 7.17-7.18 (m, 1H), 7.55 (s, 1H), 8.54 (s, 1H).

N-methyl-6-(pyridin-3-yl)pyrimidin-4-amine (21)

A mixture of **8** (0.05g, 0.35mmol), H_2O (1ml) and p-dioxane (4ml) was added the $PdCl_2dppf$ (0.03g, 0.04mmol), cesium carbonate (0.23g, 0.70mmol) and pyridine-3-boronic (0.04g, 0.35mmol) then stirred and refluxed for overnight. The reaction was quenched by saturated $NaHCO_3$(aq.) and extracted by ethyl acetate (30ml x 3). The residue was purified by flash column over silica gel (dichloromethane: methanol = 9:1, Rf = 0.44) to afford **21** (0.05g, 76.72%) as a pale yellow solid. ^{1}H-NMR (300MHz, CD_3OD): δ 2.97 (s, 3H), 6.93 (s, 1H), 7.53-7.57 (m, 1H), 8.36 (d, *J*= 8.1 Hz, 1H), 8.50 (s, 1H), 8.61-8.63 (m, 1H), 9.08 (s, 1H).

N-methyl-6-(pyridin-4-yl)pyrimidin-4-amine (22)

A mixture of **8** (0.35g, 2.44mmol), H_2O (1.5ml) and p-dioxane (6ml) was added the $PdCl_2dppf$ (0.18g, 0.24mmol), cesium carbonate (1.59g, 4.88mmol) and

pyridine-4-boronic (0.30g, 2.44mmol) then stirred and refluxed for overnight. The reaction was quenched by saturated $NaHCO_3$(aq.) and extracted by ethyl acetate (30ml x 3). The residue was purified by flash column over silica gel (ethyl acetate: n-Hexane = 1:2, Rf = 0.10) to afford **22** (0.25g, 55.02%) as a pale yellow solid. ^{1}H-NMR (500MHz, CD_3OD): δ 2.97 (s, 3H), 6.99 (s, 1H), 7.94 (s, 2H), 8.52 (s, 1H), 8.66 (d, *J*= 6.5 Hz, 2H).

1-(6-((4-(4-ethylpiperazin-1-yl)phenyl)amino)pyrimidin-4-yl)-1-methyl-3-(2,4,6-trichloro-3,5-dimethoxyphenyl)urea (23)

A mixture of 2,4,6-Trichloro-3,5-dimethoxyphenylamine (0.25g, 0.97mmol) and p-dioxane (7ml) was added the triphosgene (0.48g, 1.61mmol) dissolved in toluene (3ml) and then stirred and refluxed for overnight. The reaction was directly removed out solvent and dissolved in toluene (10ml). The mixture was added the **10** (0.25g, 0.81mmol) and stirred and refluxed for overnight. The reaction was quenched by saturated $NaHCO_3$(aq.) and extracted by ethyl acetate (30ml x 3). The residue was purified by flash column over silica gel (dichloromethane: methanol = 6:1, Rf = 0.35) to afford **23** (0.10g, 20.75%) as a pale yellow solid. ^{1}H-NMR (300MHz, $CDCl_3$): δ 1.14 (t, *J*= 7.2 Hz, 3H), 2.49 (q, *J*= 7.2 Hz, 2H), 2.63 (t, *J*= 5.1 Hz, 4H), 3.26 (t, *J*= 5.4 Hz, 4H), 3.30 (s, 3H), 3.91 (s, 6H), 6.10 (s, 1H), 6.97 (d, *J*= 9.0 Hz, 3H), 7.20 (d, *J*= 9.0 Hz, 2H), 8.34 (s, 1H), 12.76 (s, 1H).

1-(6-((4-(4-ethylpiperazin-1-yl)phenyl)amino)-2-phenylpyrimidin-4-yl)-1-methyl-3-(2,4,6-trichloro-3,5-dimethoxyphenyl)urea (24)

A mixture of 2,4,6-Trichloro-3,5-dimethoxyphenylamine (0.20g, 0.76mmol) and p-dioxane (7ml) was added the triphosgene (0.38g, 1.26mmol) dissolved in toluene (3ml) and then stirred and refluxed for overnight. The reaction was directly removed out solvent and dissolved in toluene (10ml). The mixture was added the **11** (0.25g, 0.64mmol) and stirred and refluxed for overnight. The reaction was quenched by saturated $NaHCO_3$(aq.) and extracted by ethyl acetate (30ml x 3). The residue was purified by flash column over silica gel (dichloromethane: methanol = 6:1, Rf = 0.35) to afford **24** (0.04g, 9.31%) as a pale yellow solid. ^{1}H-NMR (300MHz, $CDCl_3$): δ 1.16 (t, *J*= 7.5 Hz, 3H), 2.51 (q, *J*= 7.2 Hz, 2H), 2.66 (t, *J*= 5.1 Hz, 4H), 3.27 (t, *J*= 5.1 Hz, 4H), 3.83 (s, 3H), 3.97 (s, 6H), 6.99 (d, *J*= 9.3 Hz, 2H), 7.49-7.56 (m, 3H), 7.67 (d, *J*= 9.0 Hz, 2H), 8.48-8.51 (m, 2H), 10.72 (s, 1H).

1-(6-(4-(4-ethylpiperazin-1-yl)phenyl)pyrimidin-4-yl)-1-methyl-3-(2,4,6-trichloro-3,5-dimethoxyphenyl)urea (25)

A mixture of 2,4,6-Trichloro-3,5-dimethoxyphenylamine (0.24g, 0.92mmol) and p-dioxane (7ml) was added the triphosgene (0.45g, 1.53mmol) dissolved in toluene (3ml) and then stirred and refluxed for overnight. The reaction was directly removed out solvent and dissolved in toluene (10ml). The mixture was added the **12** (0.23g, 0.77mmol) and stirred and refluxed for overnight. The reaction was quenched by saturated $NaHCO_3$(aq.) and extracted by ethyl acetate (30ml x 3). The residue was purified by flash column over silica gel (dichloromethane: methanol = 15:1, Rf = 0.43) to afford **25** (0.07g, 15.68%) as a pale yellow solid. ^{1}H-NMR (500MHz, $CDCl_3$): δ 1.14 (t, *J*= 7.0 Hz, 3H), 2.49 (d, *J*= 7.0 Hz, 2H), 2.63 (t, *J*= 5.0 Hz, 4H), 3.38 (t, *J*= 5.0 Hz, 4H), 3.57 (s, 3H), 3.91 (d, *J*= 8.0 Hz, 6H), 7.00 (d, *J*= 9.0 Hz, 2H), 8.02 (d, *J*= 9.0 Hz, 2H), 8.83 (s, 1H), 12.59 (s, 1H).

1-methyl-1-(6-phenylpyrimidin-4-yl)-3-(2,4,6-trichloro-3,5-dimethoxyphenyl)urea (26)

A mixture of 2,4,6-Trichloro-3,5-dimethoxyphenylamine (0.25g, 0.97mmol) and p-dioxane (7ml) was added the triphosgene (0.48g, 1.61mmol) dissolved in toluene (3ml) and then stirred and refluxed for overnight. The reaction was directly removed out solvent and dissolved in toluene (10ml). The mixture was added the **13** (0.15g, 0.81mmol) and stirred and refluxed for overnight. The reaction was quenched by saturated $NaHCO_3$(aq.) and extracted by ethyl acetate (30ml x 3). The residue was purified by flash column over silica gel (dichloromethane: methanol = 9:1, Rf = 0.55) to afford **26** (0.22g, 58.07%) as a pale yellow solid. ^{1}H-NMR (300MHz, $CDCl_3$): δ 3.60 (s, 3H), 3.93 (s, 6H), 7.37 (s, 1H), 7.54-7.56 (m, 3H), 8.06-8.09 (m, 2H), 8.94 (s, 1H), 12.50 (s, 1H).

1-(6-(4-methoxyphenyl)pyrimidin-4-yl)-1-methyl-3-(2,4,6-trichloro-3,5-dimethoxyphenyl)urea (27)

A mixture of 2,4,6-Trichloro-3,5-dimethoxyphenylamine (0.25g, 0.97mmol) and p-dioxane (7ml) was added the triphosgene (0.48g, 1.61mmol) dissolved in toluene (3ml) and then stirred and refluxed for overnight. The reaction was directly removed out solvent and dissolved in toluene (10ml). The mixture was added the **14** (0.17g, 0.81mmol) and stirred and refluxed for overnight.

The reaction was quenched by saturated $NaHCO_3$(aq.) and extracted by ethyl acetate (30ml x 3). The residue was purified by flash column over silica gel (ethyl acetate: n-Hexane = 1:1, Rf= 0.45) to afford **27** (0.23g, 57.05%) as a pale yellow solid. ^{1}H-NMR (300MHz, $CDCl_3$): δ 3.60 (s, 3H), 3.90 (s, 3H), 3.93 (s, 6H), 7.04 (d, *J*= 8.7 Hz, 2H), 7.30 (s, 1H), 8.06 (d, *J*= 8.7 Hz, 2H), 8.88 (s, 1H), 12.54 (s, 1H).

1-(6-(4-fluorophenyl)pyrimidin-4-yl)-1-methyl-3-(2,4,6-trichloro-3,5-dimethoxyphenyl)urea (28)

A mixture of 2,4,6-Trichloro-3,5-dimethoxy-phenylamine (0.25g, 0.97mmol) and p-dioxane (7ml) was added the triphosgene (0.48g, 1.61mmol) dissolved in toluene (3ml) and then stirred and refluxed for overnight. The reaction was directly removed out solvent and dissolved in toluene (10ml). The mixture was added the **15** (0.16g, 0.81mmol) and stirred and refluxed for overnight. The reaction was quenched by saturated $NaHCO_3$(aq.) and extracted by ethyl acetate (30ml x 3). The residue was purified by flash column over silica gel (ethyl acetate: n-Hexane = 1:2, Rf = 0.33) to afford **28** (0.10g, 25.42%) as a pale yellow solid. ^{1}H-NMR (300MHz, $CDCl_3$): δ 3.60 (s, 3H), 3.93 (s, 6H), 7.20-7.26 (m, 2H), 7.33 (s, 1H), 8.08-8.12 (m, 2H), 8.92 (s, 1H), 12.46 (s, 1H).

1-(6-(4-chlorophenyl)pyrimidin-4-yl)-1-methyl-3-(2,4,6-trichloro-3,5-dimethoxyphenyl)urea (29)

A mixture of 2,4,6-Trichloro-3,5-dimethoxy-phenylamine (0.25g, 0.97mmol) and p-dioxane (7ml) was added the triphosgene (0.48g, 1.61mmol) dissolved in toluene (3ml) and then stirred and refluxed for overnight. The reaction was directly removed out solvent and dissolved in toluene (10ml). The mixture was added the **16** (0.15g, 0.81mmol) and stirred and refluxed for overnight. The reaction was quenched by saturated $NaHCO_3$(aq.) and extracted by ethyl acetate (30ml x 3). The residue was purified by flash column over silica gel (ethyl acetate: n-Hexane = 1:2, Rf= 0.38) to afford **29** (0.11g, 27.04%) as a pale yellow solid. ^{1}H-NMR (300MHz, $CDCl_3$): δ 3.60 (s, 3H), 3.93 (s, 6H), 7.34 (s, 1H), 7.53 (d, *J*= 8.7 Hz, 2H), 8.04 (d, *J*= 8.7 Hz, 2H), 8.92 (s, 1H), 12.44 (s, 1H).

1-(6-(4-cyanophenyl)pyrimidin-4-yl)-1-methyl-3-(2,4,6-trichloro-3,5-dimethoxyphenyl)urea (30)

A mixture of 2,4,6-Trichloro-3,5-dimethoxy-phenylamine (0.25g, 0.97mmol) and p-dioxane (7ml) was added the triphosgene (0.48g, 1.61mmol) dissolved in toluene (3ml) and then stirred and refluxed for overnight. The reaction was directly removed out solvent and dissolved in toluene (10ml). The mixture was added the **17** (0.17g, 0.81mmol) and stirred and refluxed for overnight. The reaction was quenched by saturated $NaHCO_3$(aq.) and extracted by ethyl acetate (30ml x 3). The residue was purified by flash column over silica gel (ethyl acetate: n-Hexane = 1:2, Rf= 0.23) to afford **30** (0.12g, 30.07%) as a pale yellow solid. ^{1}H-NMR (300MHz, $CDCl_3$): δ 3.61 (s, 3H), 3.93 (s, 6H), 7.40 (s, 1H), 7.84 (d, *J*= 5.4 Hz, 2H), 8.20 (d, *J*= 8.7 Hz, 2H), 8.98 (s, 1H), 12.32 (s, 1H).

1-(6-(3-cyanophenyl)pyrimidin-4-yl)-1-methyl-3-(2,4,6-trichloro-3,5-dimethoxyphenyl)urea (31)

A mixture of 2,4,6-Trichloro-3,5-dimethoxy-phenylamine (0.25g, 0.97mmol) and p-dioxane (7ml) was added the triphosgene (0.48g, 1.61mmol) dissolved in toluene (3ml) and then stirred and refluxed for overnight. The reaction was directly removed out solvent and dissolved in toluene (10ml). The mixture was added the **18** (0.17g, 0.81mmol) and stirred and refluxed for overnight. The reaction was quenched by saturated $NaHCO_3$(aq.) and extracted by ethyl acetate (30ml x 3). The residue was purified by flash column over silica gel (ethyl acetate: n-Hexane = 2:1, Rf= 0.33) to afford **31** (0.12g, 30.07%) as a pale yellow solid. ^{1}H-NMR (300MHz, $CDCl_3$): δ 3.60 (s, 3H), 3.92 (s, 6H), 7.36 (s, 1H), 7.66 (t, *J*= 8.1 Hz, 1H), 7.81 (d, *J*= 7.8 Hz, 1H), 8.30 (d, *J*= 8.4 Hz, 1H), 8.39 (s, 1H), 8.95 (s, 1H), 12.33 (s, 1H).

1-methyl-1-(6-(3-nitrophenyl)pyrimidin-4-yl)-3-(2,4,6-trichloro-3,5-dimethoxyphenyl)urea (32)

A mixture of 2,4,6-Trichloro-3,5-dimethoxy-phenylamine (0.25g, 0.97mmol) and p-dioxane (7ml) was added the triphosgene (0.48g, 1.61mmol) dissolved in toluene (3ml) and then stirred and refluxed for overnight. The reaction was directly removed out solvent and dissolved in toluene (10ml). The mixture was added the **19** (0.19g, 0.81mmol) and stirred and refluxed for overnight. The reaction was quenched by saturated $NaHCO_3$(aq.) and extracted by ethyl acetate (30ml x 3). The residue was purified by flash column over silica gel (ethyl acetate: n-Hexane = 1:2, Rf= 0.31) to afford **32** (0.06g, 14.45%) as a pale yellow solid. ^{1}H-NMR (300MHz, $CDCl_3$): δ 3.64 (s, 3H), 3.93 (s, 6H), 7.44 (s, 1H), 7.75 (t, *J*= 8.1 Hz, 1H), 8.39-8.47 (m, 2H), 8.94 (s, 1H), 8.99 (s, 1H), 12.36 (s, 1H).

1-(6-(furan-2-yl)pyrimidin-4-yl)-1-methyl-3-(2,4,6-trichloro-3,5-dimethoxyphenyl)urea (33)

A mixture of 2,4,6-Trichloro-3,5-dimethoxy-phenylamine (0.15g, 0.58mmol) and p-dioxane (7ml) was added the triphosgene (0.29g, 0.96mmol) dissolved in toluene (3ml) and then stirred and refluxed for overnight. The reaction was directly removed out solvent and dissolved in toluene (10ml). The mixture was added the **20**

(0.09g, 0.49mmol) and stirred and refluxed for overnight. The reaction was quenched by saturated $NaHCO_3$(aq.) and extracted by ethyl acetate (30ml x 3). The residue was purified by flash column over silica gel (ethyl acetate: n-Hexane = 1:4, Rf= 0.20) to afford **33** (0.05g, 22.29%) as a pale yellow solid. ^{1}H-NMR (300MHz, $CDCl_3$): δ 3.58 (s, 3H), 3.93 (s, 6H), 6.62 (d, *J*= 5.4 Hz, 1H), 7.33 (d, *J*= 6.0 Hz, 2H), 7.64 (s, 1H), 8.80 (s, 1H), 12.47 (s, 1H).

1-methyl-1-(6-(pyridin-3-yl)pyrimidin-4-yl)-3-(2,4,6-trichloro-3,5-dimethoxyphenyl)urea (34)

A mixture of 2,4,6-Trichloro-3,5-dimethoxy-phenylamine (0.25g, 0.97mmol) and p-dioxane (7ml) was added the triphosgene (0.48g, 1.61mmol) dissolved in toluene (3ml) and then stirred and refluxed for overnight. The reaction was directly removed out solvent and dissolved in toluene (10ml). The mixture was added the **21** (0.15g, 0.81mmol) and stirred and refluxed for overnight. The reaction was quenched by saturated $NaHCO_3$(aq.) and extracted by ethyl acetate (30ml x 3). The residue was purified by flash column over silica gel (dichloromethane: methanol = 9:1, Rf = 0.55) to afford **34** (0.04g, 10.54%) as a pale yellow solid. ^{1}H-NMR (300MHz, $CDCl_3$): δ 3.62 (s, 3H), 3.93 (s, 6H), 7.41 (s, 1H), 7.50-7.54 (m, 1H), 8.44 (d, *J*= 8.1 Hz, 1H), 8.79 (d, *J*= 4.2 Hz, 1H), 8.97 (s, 1H), 9.29 (s, 1H), 12.38 (s, 1H).

1-methyl-1-(6-(pyridin-4-yl)pyrimidin-4-yl)-3-(2,4,6-trichloro-3,5-dimethoxyphenyl)urea (35)

A mixture of 2,4,6-Trichloro-3,5-dimethoxy-phenylamine (0.25g, 0.97mmol) and p-dioxane (7ml) was added the triphosgene (0.48g, 1.61mmol) dissolved in toluene (3ml) and then stirred and refluxed for overnight. The reaction was directly removed out solvent and dissolved in toluene (10ml). The mixture was added the **22** (0.15g, 0.81mmol) and stirred and refluxed for overnight. The reaction was quenched by saturated $NaHCO_3$(aq.) and extracted by ethyl acetate (30ml x 3). The residue was purified by flash column over silica gel (dichloromethane: methanol = 9:1, Rf = 0.30) to afford **35** (0.20g, 52.68%) as a pale yellow solid. ^{1}H-NMR (300MHz, $CDCl_3$): δ 3.62 (s, 3H), 3.93 (s, 6H), 7.45 (s, 1H), 8.00 (d, *J*= 6.0 Hz, 2H), 8.84 (d, *J*= 4.8 Hz, 2H), 9.00 (s, 1H), 12.29 (s, 1H).

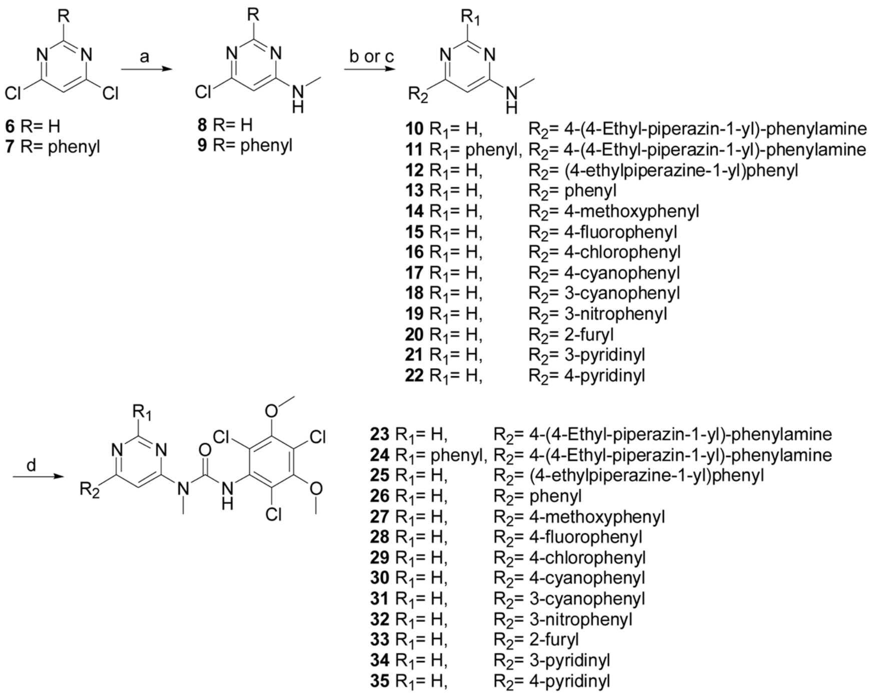

Supplementary Figure S1: The synthesis of compound 23-35. Reagents and condition. (a) 2 M Methylamine in THF, IPA, rt; (b) for **10**-**11**: 4-(4-Ethyl-piperazin-1-yl)-phenylamine, AcOH/H_2O, reflux; (c) for **12**-**22**: substituted boronic acid, $PdCl_2$(dppf), Cs_2CO_3,*p*-dioxane/H_2O, reflux; (d) 2,4,6-Trichloro-3,5-dimethoxy-phenylamine, triphosgene, *p*-dioxane, toluene, reflux then toluene, reflux. Abbreviations: THF, tetrahydrofuran; IPA, isopropyl alcohol; AcOH, acetic acid.

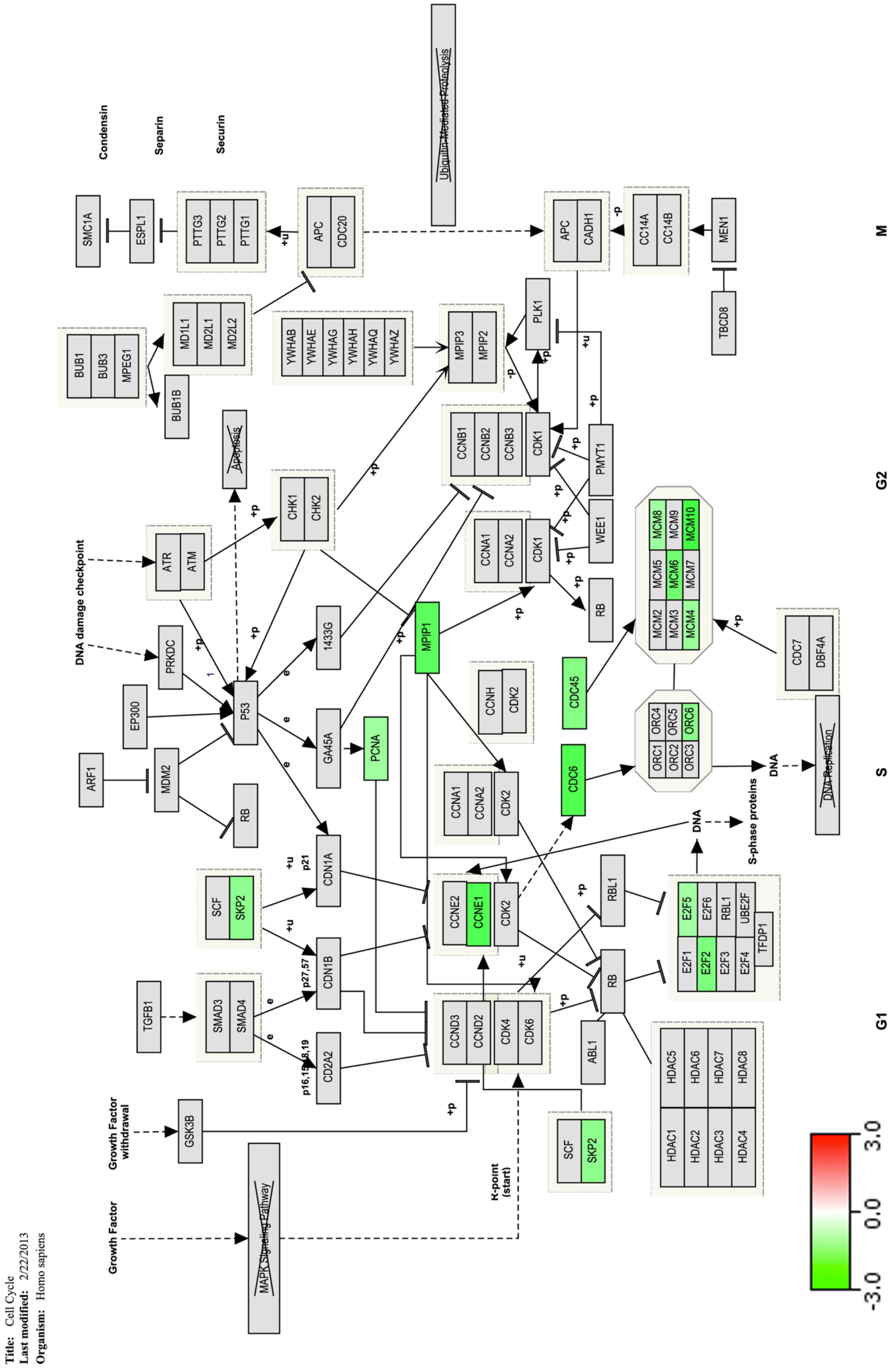

Supplementary Figure S2: MPT0L145-decreased genes associated with cell cycle. RT-112 cells were treated with MPT0L145 (4 µM) for 8 h. Total RNA was extracted and subjected to cDNA microarray analysis. Dowregulated genes associated with cell cycle progression were highlighted in green by the software PathVisio.

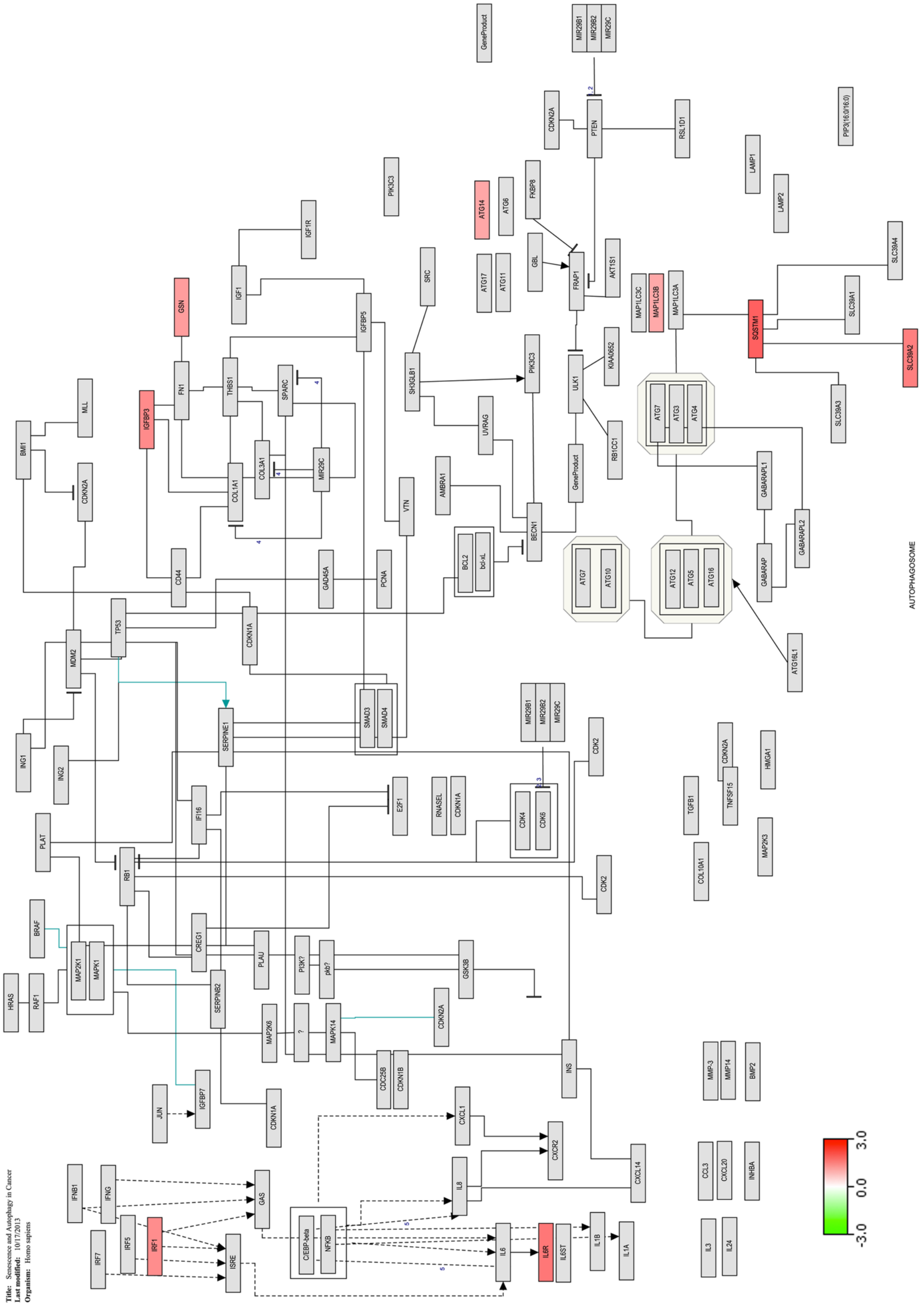

Supplementary Figure S3: MPT0L145-increased genes associated with senescence and autophagy in cancer. RT-112 cells were treated with MPT0L145 (4 μM) for 8 h. Total RNA was extracted and subjected to cDNA microarray analysis. Upregulated genes associated with senescence and autophagy were highlighted in red by the software PathVisio.

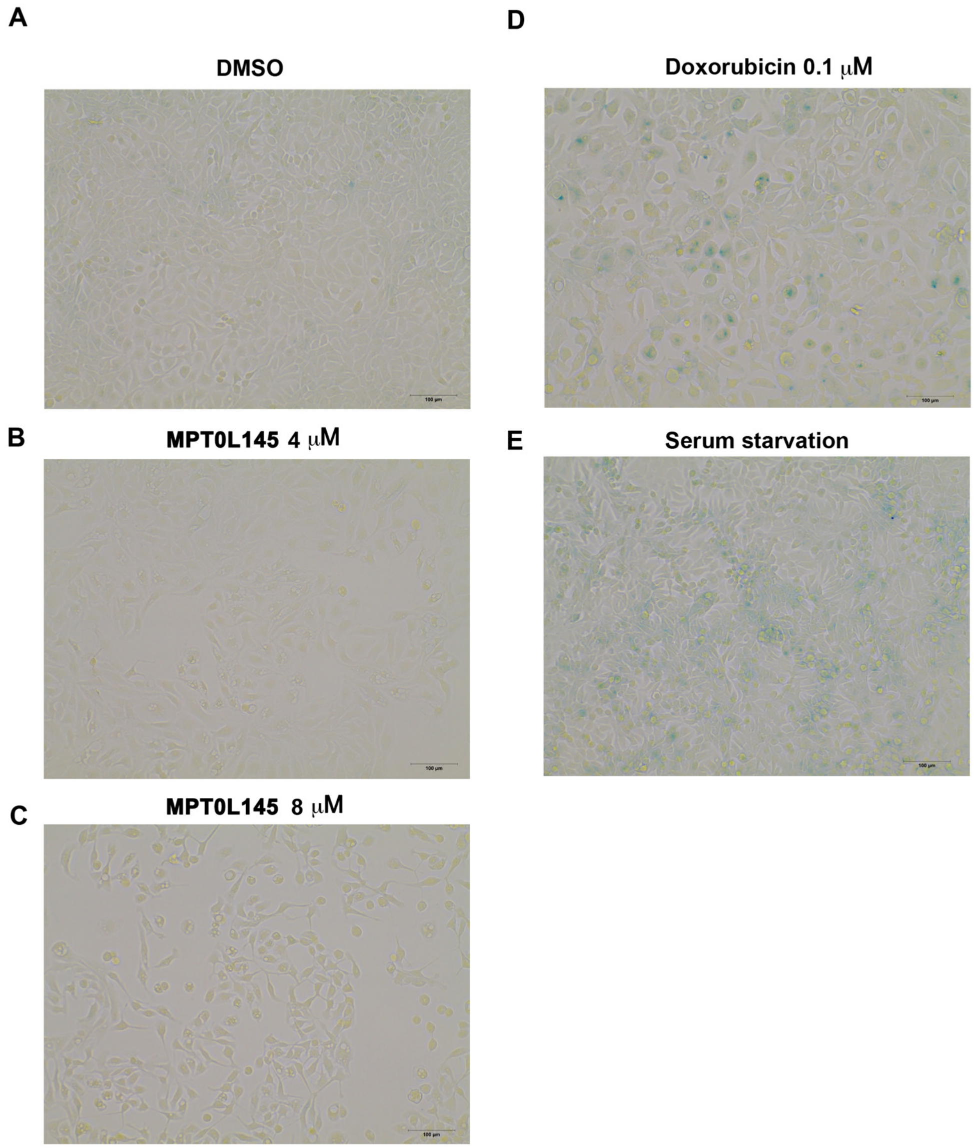

Supplementary Figure S4: Effect of MPT0L145 on senescence. RT-112 cells were treated with DMSO **A.** or MPT0L145 at 4 μM **B.** and 8 μM **C.** for 72 hours, and the cells were stained for SA-β-galactosidase activity according to manufacture's instruction (Cell Signaling Technology, Danvers, MA, USA). Doxorubicin **D.** and serum starvation **E.** were served as positive control.

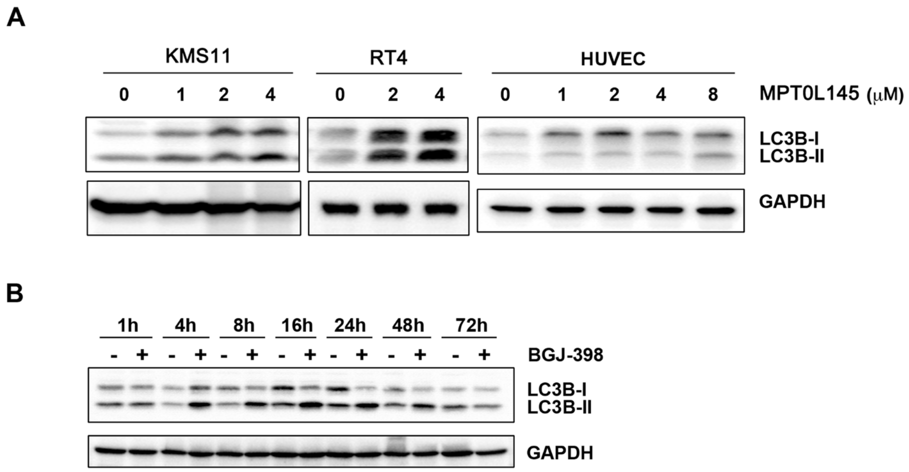

Supplementary Figure S5: Effects of FGFR inhibitors on the induction of autophagy. A. KMS11, RT4 and HUVEC cells were treated with MPT0L145 for 24 h and protein lysates subjected to western blot analysis. **B.** RT-112 cells were treated with BGJ-398 (4 μM) for the indicated times and protein lysates subjected to western blot analysis.

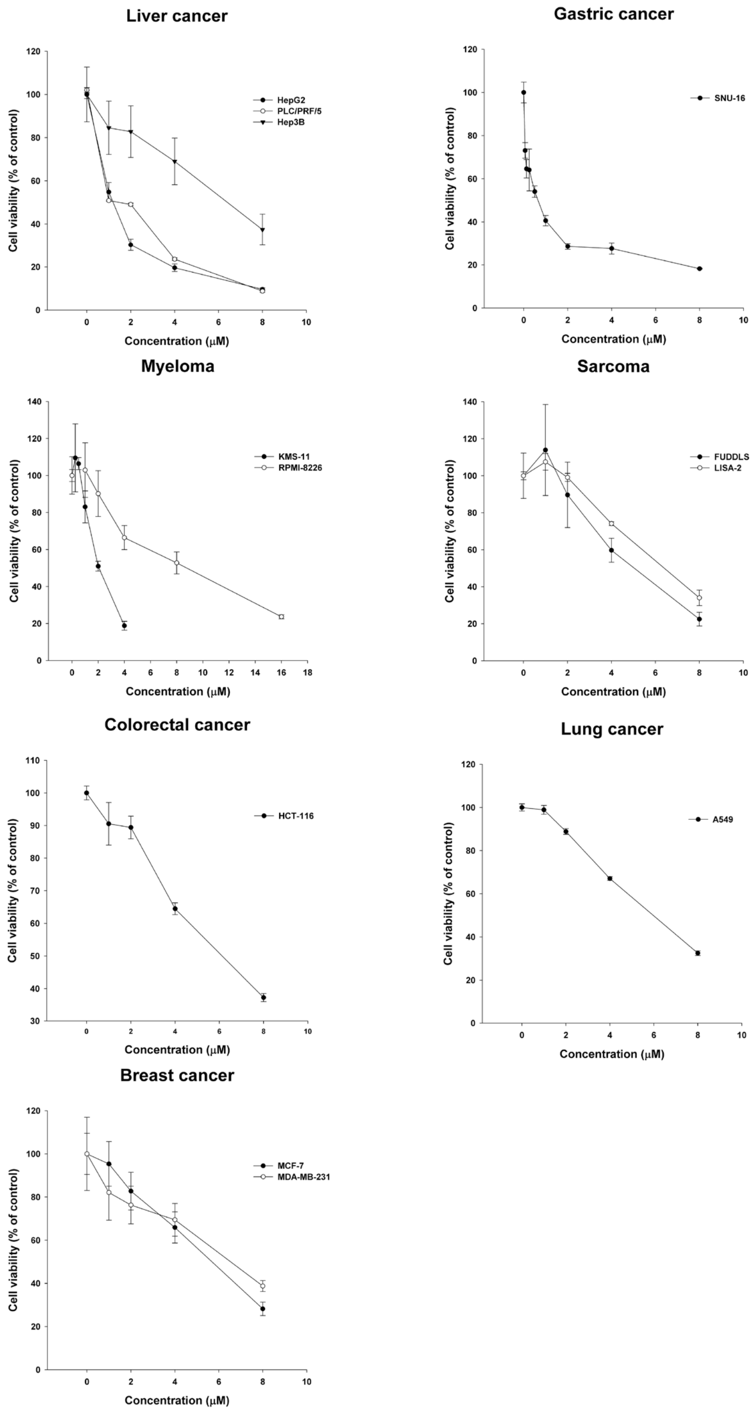

Supplementary Figure S6: Anti-growth activity of MPT0L145 in various cancer cells. Different types of cancer cell lines were treated with the indicated concentrations of MPT0L145 for 72 h. Cell viability was assessed the MTT assay. Data are expressed as means ± S.D.

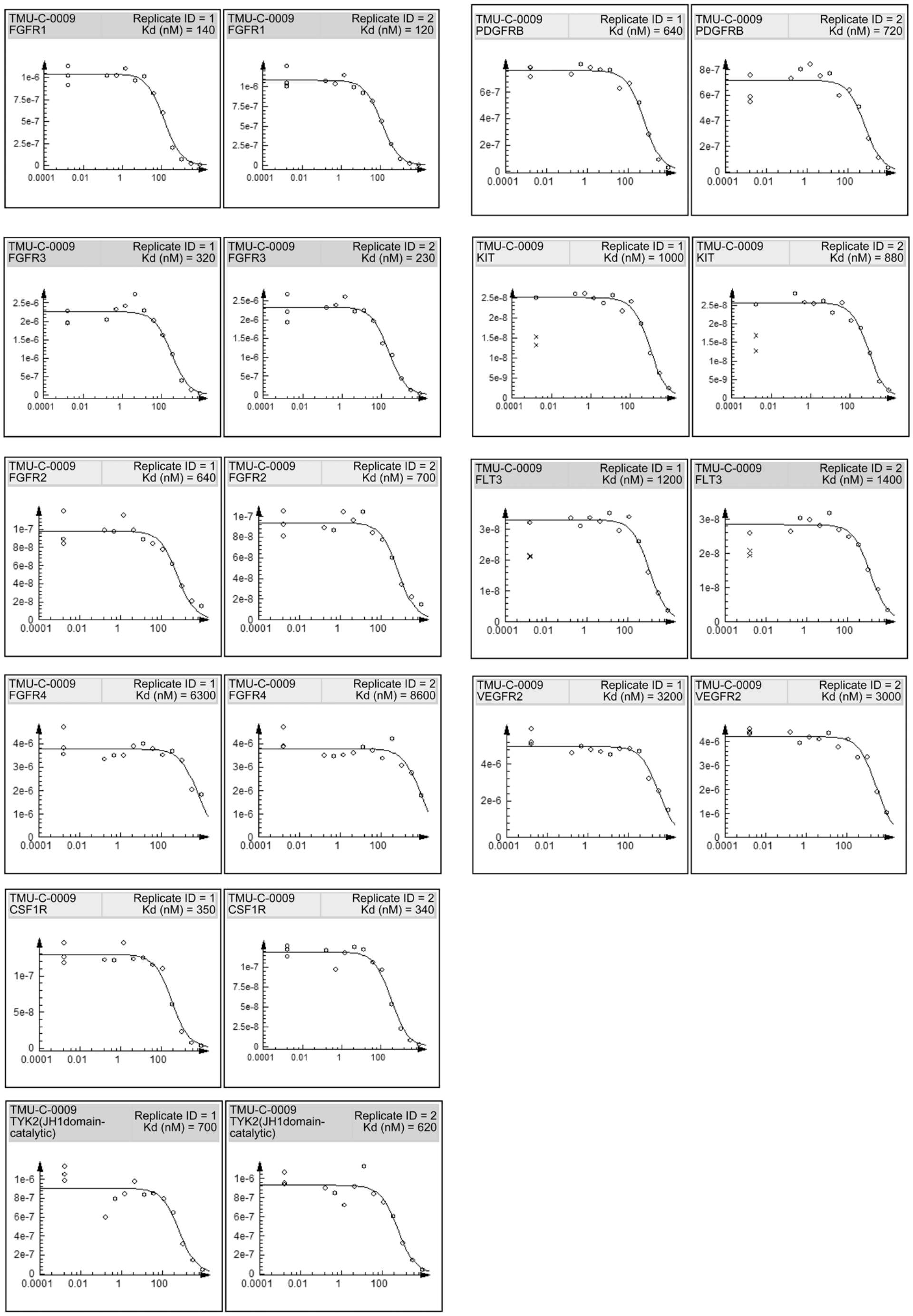

Supplementary Figure S7: *In vitro* inhibitory effects of MPT0L145 on a panel of protein kinases. The inhibitory activities of MPT0L145 on protein kinases were assessed by the service of KINOMEscan® (DiscoverRx, Fremont, CA, USA). The screening is based on a competition-binding assay and the Kd values in duplicates were shown.

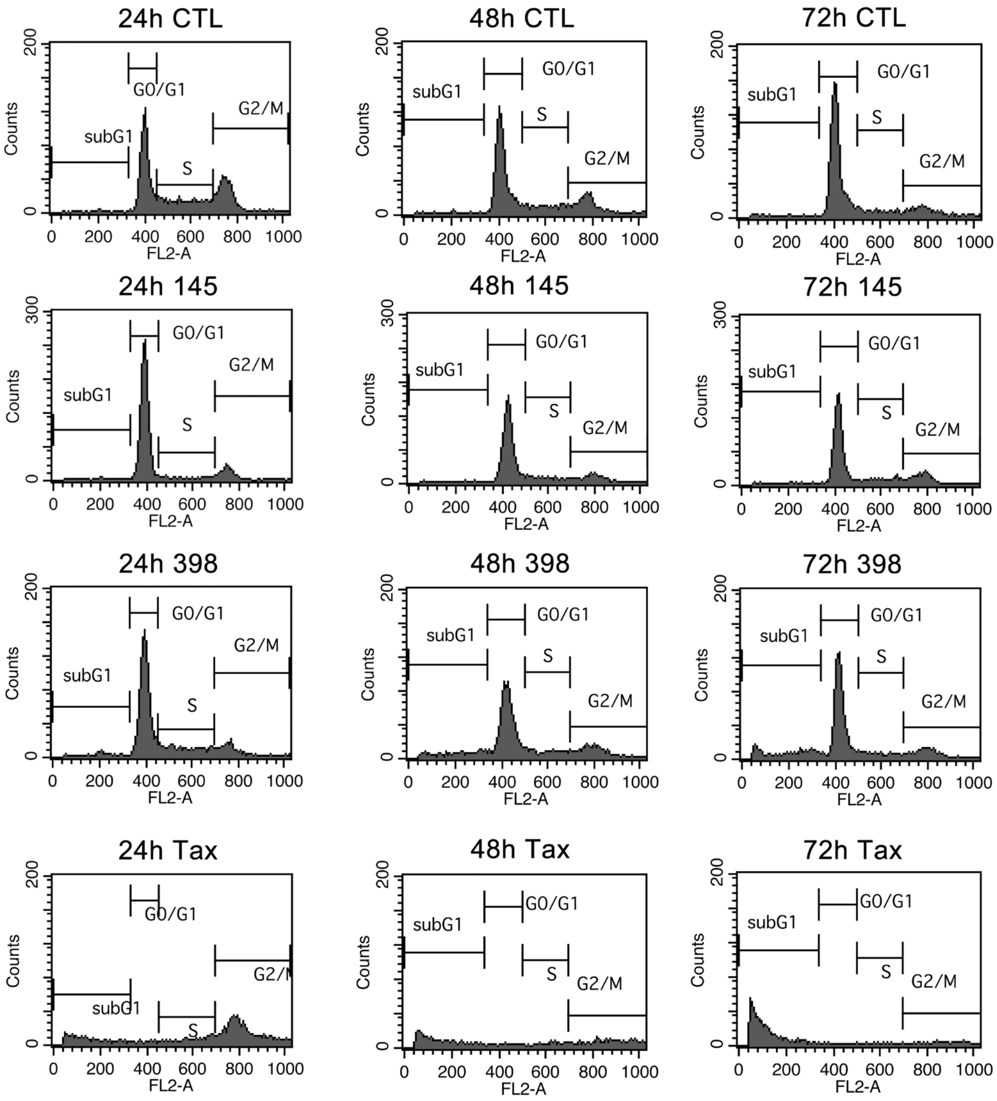

Supplementary Figure S8: The effects of MPT0L145 on cell cycle distribution in RT-112 cells. RT-112 cells were treated with MPT0L145 (4 μM), BGJ-398 (4 μM) and Paclitaxel (0.1 μM) for the indicated times, and cell cycle distribution was analyzed via flow cytometry. (CTL: control group, 145: MPT0L145, 398: BGJ-398, Tax: Paclitaxel)

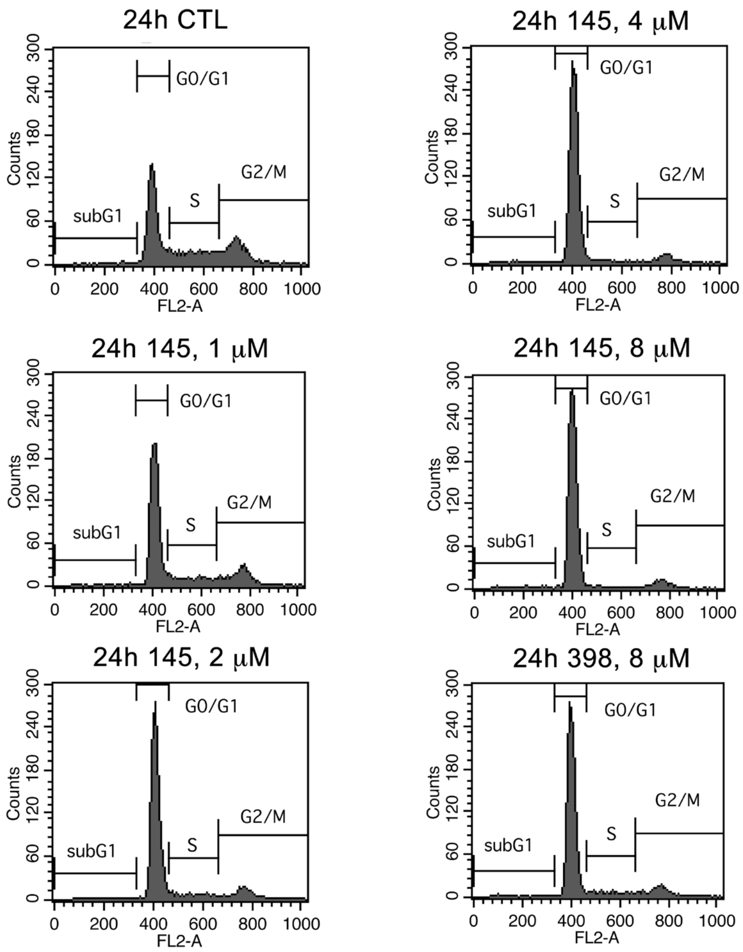

Supplementary Figure S9: MPT0L145 induced G_0/G_1 arrest in RT-12 cells. RT-112 cells were exposed to various concentrations of MPT0L145 and BGJ-398 for 24 h, and cell cycle distribution was analyzed via flow cytometry. (CTL: control group, 145: MPT0L145, 398: BGJ-398)

Table S1: Anti-proliferative activity of compound 1, 23-35

		Viability (% of control cells)									
		RT-112 (10 μM)		RT-112 (5 μM)		Hep3B (5 μM)		PLC5/ PRF/5 (5 μM)		SNU-16 (5 μM)	
Code	Compound No.	Mean	S.D.	Mean	S.D.	Mean	S.D.	Mean	S.D.	Mean	S.D.
MPT0L145	**1**	8.5	0.7	33.4	7.5	66.2	6.4	20.6	1.1	36.9	7.9
MPT0L146	**24**	29.7	3.8	100.8	5.8	94.3	1.2	88.0	0.4	83.4	13.3
MPT0L090	**23**	12.7	1.0	52.6	5.3	47.1	6.5	72.6	3.8	27.5	0.7
MPT0L182	**25**	8.6	2.0	95.6	3.7	96.3	4.7	78.3	7.8	63.2	4.6
MPT0L149	**26**	82.3	1.3								
MPT0L153	**27**	43.0	3.4	63.8	13.9	93.6	6.6	91.3	3.6	76.2	2.4
MPT0L155	**28**	44.4	2.2	92.6	1.6	72.8	1.1	55.3	3.9	80.5	6.1
MPT0L156	**29**	34.8	1.5	98.3	2.2	54.3	3.1	44.4	4.8	76.7	8.1
MPT0L152	**30**	55.3	1.2								
MPT0L150	**31**	75.0	4.3								
MPT0L151	**32**	63.8	0.8								
MPT0L154	**33**	76.1	8.0								
MPT0L147	**34**	85.0	2.7								
MPT0L148	**35**	89.3	3.5								

Table S2: *In vitro* inhibitory effects of MPT0L145 on a panel of protein kinases

Kinase	K_d (nM)	Kinase	K_d (nM)
FGFR1	130	IKK-alpha	>10000
FGFR3	270	IKK-beta	>10000
FGFR2	670	INSR	>10000
FGFR4	7500	KIT	>10000
CSF1R	340	LKB1	>10000
TYK2	660	MAPKAPK2	>10000
PDGFRB	680	MEK1	>10000
KIT	950	MEK2	>10000
FLT3	1300	MET	>10000
VEGFR2	3100	MKNK1	>10000
Erbb2	10000	MKNK2	>10000
ACVR1B	>10000	MLK1	>10000
ADCK3	>10000	p38-alpha	>10000
AKT1	>10000	p38-beta	>10000
AKT2	>10000	PDGFRA	>10000
AURKB	>10000	PDPK1	>10000
AXL	>10000	PIK3CA	>10000
BMPR2	>10000	PIK3CG	>10000
BRAF	>10000	PIM1	>10000
BTK	>10000	PIM2	>10000
CDK3	>10000	PIM3	>10000
CDK9	>10000	PLK1	>10000
CSNK1D	>10000	PLK3	>10000
DCAMKL1	>10000	RAF1	>10000
EGFR	>10000	RET	>10000
EPHA2	>10000	ROCK2	>10000
ERBB4	>10000	SRPK3	>10000
ERK1	>10000	TGFBR1	>10000
FAK	>10000	TSSK1B	>10000
GSK3B	>10000	YANK3	>10000
IGF1R	>10000	ZAP70	>10000

Table S3. ***In vitro*** **antiproliferative activity of MPT0L145 in a panel of cancer cell lines**

Cancer type	Cell name	IC_{50} (μM)	FGFR status (Reference)
Bladder	RT-112	1.63±0.01	FGFR3 overexpression (16)<br>FGFR3-TACC3 fusion (40)
Bladder	RT4	4.00±0.58	FGFR3 overexpression (16)<br>FGFR3-TACC3 fusion (40)
Bladder	T24	6.00±0.50	
Liver	HepG2	1.18±0.16	FGFR3 and FGFR4 (19)<br>FGFR3-TACC3 fusion (19)
Liver	PLC/PRF/5	1.48±0.30	FGFR1 and FGFR4 (19)
Liver	Hep3B	6.47 ±0.38	
Gastric	SNU-16	0.65 ± 0.07	FGFR2 overexpression (7)
Myeloma	KMS-11	2.05 ±0.11	FGFR3 mutation (Y373C) (8)
Myeloma	RPMI-8226	8.51±1.77	
Sarcoma	FUDDLS	4.74 ±0.49	
Sarcoma	LISA-2	6.43±0.26	
Colorectal	HCT-116	6.12±0.01	
Lung	A549	5.97±0.10	
Breast	MCF-7	5.98±0.68	
Breast	MDA-MB-231	6.04 ±0.26	
Normal cell	HUVEC	11.1±2.28	
